# Pharmaceuticals targeting signaling pathways of endometriosis as potential new medical treatment: A review

**DOI:** 10.1002/med.21802

**Published:** 2021-05-05

**Authors:** Sze Wan Hung, Ruizhe Zhang, Zhouyurong Tan, Jacqueline Pui Wah Chung, Tao Zhang, Chi Chiu Wang

**Affiliations:** ^1^ Department of Obstetrics and Gynaecology The Chinese University of Hong Kong Hong Kong; ^2^ Center for Reproductive Medicine, Henan Key Laboratory of Reproduction and Genetics The First Affiliated Hospital of Zhengzhou University Zhengzhou; ^3^ Reproduction and Development, Li Ka Shing Institute of Health Sciences The Chinese University of Hong Kong Hong Kong; ^4^ School of Biomedical Sciences The Chinese University of Hong Kong Hong Kong; ^5^ Chinese University of Hong Kong‐Sichuan University Joint Laboratory in Reproductive Medicine The Chinese University of Hong Kong Hong Kong

**Keywords:** endometriosis, pathophysiology, pathways, pharmaceuticals, targets, treatments

## Abstract

Endometriosis (EM) is defined as endometrial tissues found outside the uterus. Growth and development of endometriotic cells in ectopic sites can be promoted via multiple pathways, including MAPK/MEK/ERK, PI3K/Akt/mTOR, NF‐κB, Rho/ROCK, reactive oxidative stress, tumor necrosis factor, transforming growth factor‐β, Wnt/β‐catenin, vascular endothelial growth factor, estrogen, and cytokines. The underlying pathophysiological mechanisms include proliferation, apoptosis, autophagy, migration, invasion, fibrosis, angiogenesis, oxidative stress, inflammation, and immune escape. Current medical treatments for EM are mainly hormonal and symptomatic, and thus the development of new, effective, and safe pharmaceuticals targeting specific molecular and signaling pathways is needed. Here, we systematically reviewed the literature focused on pharmaceuticals that specifically target the molecular and signaling pathways involved in the pathophysiology of EM. Potential drug targets, their upstream and downstream molecules with key aberrant signaling, and the regulatory mechanisms promoting the growth and development of endometriotic cells and tissues were discussed. Hormonal pharmaceuticals, including melatonin, exerts proapoptotic via regulating matrix metallopeptidase activity while nonhormonal pharmaceutical sorafenib exerts antiproliferative effect via MAPK/ERK pathway and antiangiogenesis activity via VEGF/VEGFR pathway. N‐acetyl cysteine, curcumin, and ginsenoside exert antioxidant and anti‐inflammatory effects via radical scavenging activity. Natural products have high efficacy with minimal side effects; for example, resveratrol and epigallocatechin gallate have multiple targets and provide synergistic efficacy to resolve the complexity of the pathophysiology of EM, showing promising efficacy in treating EM. Although new medical treatments are currently being developed, more detailed pharmacological studies and large sample size clinical trials are needed to confirm the efficacy and safety of these treatments in the near future.

## 1 INTRODUCTION

### 1.1 Epidemiology and pathogenesis of endometriosis (EM)

EM is a disease caused by functional endometrial tissues growing in other areas outside the uterine cavity. It is a chronic disease that affects productivity and quality of life in women.^1^ The typical presenting symptoms in women with EM include chronic pelvic pain, abnormal menstruation, and dyspareunia. EM occurs frequently in women of reproductive age, and the incidence is approximately 10%.^2^ Approximately 40%–60% of women with EM experience dysmenorrhea, and 20%–30% are complicated with infertility.^3^

Although EM presents as benign clinical and pathological manifestations, it has similar characteristics to cancers, including dissemination, invasion, and hyperplasia. It is generally accepted that EM is a hormone‐dependent disease.^4^ Estrogen (E_2_) augmentation and progesterone resistance feature EM pathology, but the mechanism of how this occurs is unclear. Nevertheless, EM has been observed even in the absence of increased E_2_ production in postmenopausal women.^5^ The pathogenesis of EM is dominated by the theory of ectopic implantation of the endometrium, along with multiple factors, such as endocrine, immunity, invasion, and angiogenesis. Retrograde menstruation theory suggests reflux of endometrial tissue through the fallopian tubes during menstruation and implantation into the peritoneal cavity.^6^, ^7^ Lymphatic and vascular dissemination theories suggest that endometrial cells disseminate via lymphatic or blood circulation.^8^ Stem cell origin theory suggests that undifferentiated peritoneal tissue, ovarian surface epithelial tissue, and endometrium mesenchymal stem cells transform into endometrial‐like tissue in response to retrograde menstrual blood flow and stimulation from chronic inflammatory factors.^9^

EM development is also associated with a combination of genetic variation and environmental factors. First‐degree relatives of women with EM have a seven fold greater risk of developing EM than those without a family history, and the risk of developing the disease in identical twins of women with EM is as high as 75%.^10^, ^11^ In recent years, the increased incidence of EM is also thought to be associated with exposure to environmental pollutants. Tetrachlorodibenzo‐p‐dioxin (TCDD) is the most prevalent air pollutant worldwide, and it promotes cytokine secretion. Endogenous E_2_ exacerbates the effects of TCDD and the interaction of the two chemicals provokes inflammatory responses, induces toxicity, and thus increases the severity of EM.^12^, ^13^, ^14^ Therefore, the pathophysiology of EM is complex, interrelated, and specific, thereby requiring multiple targeted therapies.

### 1.2 Dysregulated molecular and signaling pathways

Regardless of EM theories, endometrial cells must complete a serial process of immune escape, survival, adhesion, invasion, and angiogenesis to develop and grow in the ectopic sites.^15^ Signaling pathway refers to a series of enzymatic reaction pathways that pass molecular signals into cells through the cell membrane to exert corresponding effects. EM‐related signaling pathways, together with their upstream and downstream regulatory factors, constitute a large and complex transduction system and play an important role in the occurrence and development of EM. Abnormalities in these pathways and their interactions can lead to abnormal proliferation, apoptosis, autophagy, adhesion, invasion, fibrosis, angiogenesis, reactive oxidative stress (ROS), immune system, and inflammatory responses of the ectopic endometrial tissues, thereby promoting its growth and development. Hormonal‐related enzymes, growth factors, inflammatory cytokines and chemokines, such as tumor necrosis factor (TNF)‐α, transforming growth factors (TGF)‐β, prostaglandin E_2_ (PGE_2_), prostaglandin‐endoperoxide synthase (COX)2 play important roles in these processes.^16^, ^17^ They induce local immune imbalance in the microenvironment to tolerate immune clearance and promote the survival of ectopic lesions. Downstream molecules, such as hypoxia‐inducible factors (HIF)‐1α, matrix metallopeptidase (MMPs), and vascular endothelial growth factors (VEGFs), are dysregulated and play roles in the angiogenesis and growth of EM lesions.^2^, ^15^, ^16^, ^17^

### 1.3 Current treatment of EM

Current treatments for EM include surgical and medical therapies. Conservative surgery removes the EM deposits but increases the risk of impairing ovarian reserve, harming other organs, and imposing postoperative recurrence.^18^ Therefore, medical therapy (Table 1) always comes first into consideration, and the choices depend on multiple factors, such as symptom severity, conceive desire, and comorbidities. Generic classes of medical therapies for EM include hormonal therapy, including oral contraceptives (COC), progesterone and gonadotropin‐releasing hormone (GnRH) agonist and antagonist, and nonhormonal therapies such as nonsteroidal anti‐inflammatory drugs (NSAIDs).

**Table 1:** Current FDA‐approved medication for endometriosis treatment

**Medication**	**Generic name**	**Rank**	**Market name** a	**Price range** a b	**Administration** a	**Mechanism of action**	**Advantages**	**Disadvantages**	**Reference**
Combinations of ethinyl estradiol + norgestimate (3rd‐generation progestin)	Estrogen and progestin (COC)	1st line^c^	Previfem	$	Oral tablet	Suppresses ovarian activity, and reduces estrogen‐induced production of prostaglandins and inflammation.	Tolerable side effects. Cost‐effective. Combined use of progestin with ethinyl estradiol reduces adverse effects such as thromboembolism.	Side effects related to hypoestrogenism, such as hot flashes, dry vagina, nausea, headaches, and so forth. Adverse effect associated with long‐term usage such as thromboembolism and stroke. High recurrence rate after discontinuation. Risk of impaired fertility	[19, 20]
			Tri‐Previfem						
			Sprintec						
			Tri‐Sprintec						
			Estarylla						
			Mono‐Linyah						
			Tri‐Lo‐Sprintec						
			Tri‐Estarylla						
			Tri‐Linyah						
			Tri‐Lo‐Marzia						
Combinations of ethinyl estradiol + norethindrone (1st‐generation progestin)			Femhrt (Jinteli)	$$					
			Jevantique Lo (Fyavolv)						
Combinations of estrogen + ethynodiol diacetate (1st‐generation progestin)			Zovia 1/35E	$					
			Zovia 1/50E						
			Kelnor 1/50						
			Kelnor 1/35						
Progestin	NETA (1st‐generation progestin)	2nd line	Camila	$$	Oral tablet	Multiple mechanisms of actions that include 1. suppress ovulation and E_2_ level results in endometrial thinning, 2. Induce endometrium decidualization and inhibit estrogen, results in atrophy of lesions.	Available in different forms of administration and in different price ranges. Intramuscular injection form of treatments avoids daily administration and reduces gastrointestinal absorption. High specificity and minimal side effects with Dienogest.	Side effects related to hypoestrogenism, such as hot flashes, dry vagina, nausea, headaches, and so forth. Adverse effect associated with long‐term usages such as reduction in bone mineral density and virginal bleeding.	[21, 22]
			Nora‐Be						
			Ortho Micronor						
			Errin						
			Jolivette						
			Sharobel						
			Jencycla						
			Deblitane						
			Incassia						
			Norlyda						
			Norlyroc						
			Heather						
			Lyza						
			Aygestin	$$$					
	Medroxyprogesterone acetate	2nd line	Depo‐Provera	$$	Intramuscular injection				
			Provera	$$	Oral tablet				
	Dienogest (4th‐generation progestin)	2nd line	Visanne	$$	Oral tablet				
	Levonorgestrel‐Releasing Intrauterine Device	2nd or 3rd line	Mirena	$$$	Intrauterine system				
GnRH agonist	Nafarelin acetate	2nd or 3rd line	Synarel	$$$$$	Nasal Spray	Initial pituitary flare effect results in stimulation of pituitary LH and FSH, that deregulates pituitary GnRH receptor, suppresses pituitary secretion of LH and FSH, suppresses ovulation, mimic menopause sate and results in low circulating E_2_ and P4, leads to shrinkage of endometrium.	Available in different forms of administration and in different price ranges. Direct effect on endometriotic tissues. Approved add‐back therapy can reduce side effects.	Side effects related to hypoestrogenism, such as hot flashes, dry vagina, nausea, headaches, and so forth. Aromatase inhibitors need to be taken to prevent initial pituitary flare effect.	[22]
	Goserelin		Zoladex	$$$	subcutaneous injection				
17‐ethinyl testosterone derivative	Danazol	NA	Danocrine	$$$$	Oral tablet	Increases testosterone levels to inhibit pituitary gonadotropin secretion and E_2_ production by P450AROM and other enzymes.	Long history—First approved drug for EM.	Side effects related to hyperandrogenism such as hirsutism and muscle cramps. Increased risk of ovarian cancer. Replaced by alternative agents due to adverse effects.	[22, 23]
GnRH antagonist	Elagolix	NA	Orilissa	$$$$	Oral tablet	Directly blocks GnRH receptor on the pituitary gland to rapidly suppresses FSH, LH, and gonadal sex steroid production.	Lower degree of hypoestrogenism side effects compared to GnRH agonists. Flexible and rapid reversible onset and offset.	Side effects related to hypoestrogenism, such as hot flashes, dry vagina, nausea, headaches, and so forth. Adverse effect associated with long‐term usage such as reduction in bone mineral density.	[24, 34]
LHRH agonist	leuprolide	NA	Lupron Depot	$$$$$	Intramuscular injection	Overstimulates production of LH and disrupt endogenous hormonal feedback systems to reduce LH and gonadal sex steroid production.	Convenient as one injection per month.	Side effects related to hypoestrogenism, such as hot flashes, dry vagina, nausea, headaches, and so forth. Adverse effect associated with long‐term usage such as osteoporosis. Expensive	[25]
Combinations of LHRH agonist and progesterone (1st‐generation progestin)	Leuprolide and Norethindrone	NA	Lupaneta Pack	$$$$	Intramuscular injection	Suppresses Gonadotrope secretion of LH and FSH to reduce gonadal sex steroid production.	Combined use of Leuprolide with Norethindroe prevents bone thinning. Convenient as one injection per month.	Side effects related to hypoestrogenism, such as hot flashes, dry vagina, nausea, headaches, and so forth.	[25]

Abbreviations: COC, combined oral contraceptive; E_2_, estradiol; FDA, Food and Drug Administration; FSH, follicle‐stimulating hormone; GnRH, gonadotropin‐releasing hormone; LH, luteinizing hormone; LHRH, luteinizing hormone‐releasing hormone; NETA, norethindrone acetate; NSAID, nonsteroidal anti‐inflammatory drug; P4, progesterone; P450AROM, aromatase.

^a^ Data was extracted from The Drugs.com Database, drugs.com.

^b^ Price range was justified based on 3‐months therapy. $ denotes the approximate price range and are labelled as follows, $ (<$100); $$ ($100–$499); $$$ ($500–$1999); $$$$ ($2000–$4999); $$$$$ (>$5000).

^c^ Medication is usually prescribed together with NSAIDs.

The available reports on the effectiveness of NSAIDs on pain relief in EM are very limited, and there is no strong evidence to support a conclusion.^1^ Among all medical treatments, combined COC and progestin monotherapy represent the first‐line therapy, which can be applied to most women clinically diagnosed with EM with or without a surgical diagnosis.^26^ Continuous COC effectively reduces the recurrence of dysmenorrhea,^27^ and progestin suppresses ovulation by maintaining a hypoestrogenic state. Women with risk factors such as thrombosis and myocardial infarction may tolerate the side effects of progestin better than those of COC.^28^ To date, few derivatives of progesterone, namely, depot medroxyprogesterone acetate and norethindrone acetate, have been approved by the US Food and Drug Administration (FDA) as the sole therapy for EM.^29^, ^30^

Although GnRH is an effective hormonal treatment for EM, severe hypoestrogenic symptoms limit long‐term compliance.^31^, ^32^ GnRH agonists are second‐line hormonal therapies that exert strong action on the GnRH receptor, leading to an initial short stimulation and subsequent suppression of gonadotropin secretion. Decreased hormone levels result in the dormancy of endometriotic lesions. Owing to its long‐term adverse effects, especially osteoporosis, an add‐back therapy is recommended.^33^ Recently, the FDA approved elagolix, a nonpeptide small molecule GnRH receptor antagonist that suppresses luteinizing hormone and follicle‐stimulating hormone and correspondingly reduces E_2_ and progesterone, as a treatment for moderate to severe EM‐associated pain. Its efficacy was shown after a 6‐month treatment, but it also caused a significant decrease in bone mineral density as the main side effect.^34^ To overcome EM refractory to current hormonal treatments and NSAIDs, there have been extensive research of new medicines in recent years. Other than therapeutic efficacy, the potential use of a drug as a preventive treatment after surgery is also desirable. The recurrence of EM and the associated symptoms within 5 years after laparoscopy is approximately 19% in patients with endometrioma,^35^ and up to 10% of women require secondary surgery after 1 year,^36^ emphasizing the need for new medical treatments to prevent a recurrence.

**Figure 1:**
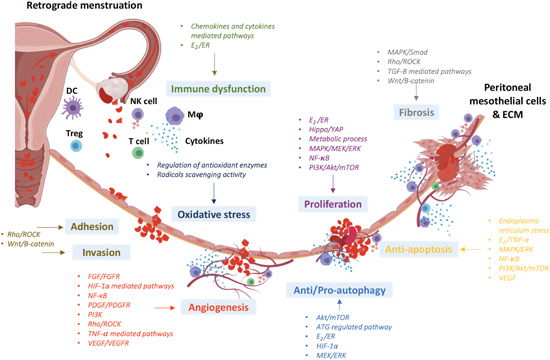
Pathophysiology of endometriosis. The schematic diagram was created using BioRender.com. Akt, protein kinase B; ATG, autophagy‐related genes; DC, dendritic cells; E_2_, estrogen; ECM, extracellular matrix; ER, estrogen eceptor; ERK extracellular signal‐regulated kinase; FGF, fibroblast growth factor; FGFR, fibroblast growth factor receptors; HIF, hypoxia‐inducible factors; MΦ, macrophages; MAPK, mitogen‐activated protein kinase; MEK, ERK kinase; mTOR, mammalian target of rapamycin; NF‐κB, nuclear factor κB; NK, natural killer; PDGF, platelet‐derived growth factor; PDGFR, platelet‐derived growth factor receptor; PI3K, phosphoinositide 3‐kinases; Rho, Ras homolog family; ROCK, Rho‐associated coiled‐coil kinase; VEGF, vascular endothelial growth factor; TGF, transforming growth factor; TNF, tumor necrosis factor; Treg, regulatory T cells; Wnt, wingless‐type mouse mammary tumor virus integration site family; YAP, Yes‐associated protein

In summary, to identify and develop new pharmaceuticals for EM treatment, understanding the dysregulated molecular and signaling pathways in EM development is essential (Figure 1). Numerous studies have focused on the antiproliferation mechanism and related targeted therapies in EM models and/or endometrial cells.^37^, ^38^ Owing to the interaction of different signaling pathways, the efficacy of potential pharmaceuticals in promoting or inhibiting a single signaling pathway is often very limited. Therefore, pharmaceutical targeting multisignaling pathways in EM has become important in the medical treatment of EM. An overview of the molecular pathways involved in the pathophysiology of EM has been reported by various publications,^39^, ^40^, ^41^ which provides a high quality evidence of the underlying pathophysiology of EM. However, previous publications only focused on currently available pharmaceuticals. In this review, we aimed to present an updated summary of studies focusing on new potential pharmaceuticals, including preclinical studies, clinical trials, as well as studies on marketed pharmaceuticals. In‐depth studies of signaling pathways targeted by pharmaceuticals are currently an emerging research direction, which will open up broad prospects for the new generation of EM treatment.

## 2 DEFINITION OF POTENTIAL PHARMACEUTICALS FOR EM TREATMENT

### 2.1 Experimental evidence in vitro and in vivo

The choice of investigation models considerably influences the translational potential of preclinical research. Endometriotic and endometrial tissue cells with specific cell characteristics, defined by their morphology and phenotypes, confirmed by immunocytochemistry allow in vitro investigations of the mechanism of hormonal expression, cytokine secretion, cell proliferation, and differentiation.^42^ Romano et al.^43^ critically analyzed different EM culture models of samples from peritoneal, ovarian, and deep infiltration EM and recommended a guideline for assessing the quality of both primary endometriotic cells and immortalized endometriotic cell lines. Culture conditions can imitate EM in situ; for example, endometrium undergoing menstruation,^44^ macrophage activation,^45^ epithelium mesothelium transformation,^46^ and cell–cell interactions.^47^, ^48^ In addition, in vivo animal experiments provide a biological system with an integrative environment and complete cellular and molecular network for lesion development and growth in vivo. It mimics the conditions in humans in the hopes that the results can be translated from bench to clinic. The application and limitations of various EM animal models, including autotransplantation of uterine tissues and xenotransplantation of human endometrial tissues into the peritoneal cavity or subcutaneous pocket in ectopic sites of rodent models, as well as in the primate model have been assessed, and the choice of the appropriate model for studies depends on the research questions.^49^

Apart from the appropriate model, positive control of current pharmaceuticals should be included for comparison, which will serve as experimental evidence of the efficacy of new drugs. When choosing a positive control, pharmaceuticals with relevant actions to the examined molecular and signaling pathways should be considered. For example, dienogest can be used as a positive control to compare the inhibition of NF‐κB activation, enhancement of apoptosis, or inhibition of MMP‐2/‐9,^50^, ^51^ leuprolide acetate to compare the inhibition of promitogenic cytokines,^52^ and celecoxib to compare the proliferation‐inhibitory and apoptosis‐enhancing effects.^53^

### 2.2 Pharmacokinetics, pharmacodynamics, and safety profile

In addition to the efficacy, the pharmacokinetic profile of a drug with respect to absorption, distribution, metabolism, and excretion should be available to support its clinical use.^54^ The bioavailability of a drug and its active metabolites in systematic circulation and local tissues should be quantified to justify the therapeutic dosage for clinical application.^55^ The relationship between drug potency and pharmacological effects on the body and action site should be evaluated to prevent off‐target toxicities.^56^ The possible adverse effects on other tissues also need to be determined. Medications with specific efficacy on the ectopic endometrium and minimal side effects on the eutopic endometrium are preferable for EM treatment, as these medications will affect reproductive cycles the least. In animal experiments, adverse effects on reproductive tissues and functions should be carefully monitored. As a short‐term measure, no significant change in body weight should occur in the test animals, and as a long‐term measure, the animals should be able to conceive and deliver. For women with EM who prefer symptomatic medical therapy, such side effects should be limited and well‐tolerated. Medicines that regulate E_2_ levels usually result in hypoestrogenism and are associated with side effects such as hot flushes and vaginal dryness, which are acceptable, but not preferable.^57^ Other common adverse effects, such as osteoporosis and venous thromboembolism, should be avoided. The effect of the medications on fertility should also be monitored; however, the current data are very limited.

### 2.3 Cost‐effectiveness analysis

Several studies have systematically recorded the direct and indirect costs of EM treatment and highlighted its long‐term economic burden on the society, healthcare system, and affected women.^58^ This has raised awareness of the disease and increased the demand for cost‐effective EM drugs. However, the choice of treatment depends not only on patients’ desired outcomes but also on treatment affordability. A cost‐effective medication is that with equivalent monetary value and efficiency. Therefore, as the most cost‐effective treatment for EM is considered for use as a standard hormonal treatment,^59^ a potential new pharmaceutical should be affordable and easily accessible to the market, in addition to showing good efficacy with fewer side effects.

In summary, a potential new pharmaceutical should be well‐studied in terms of not only action mechanism and efficacy *in vitro* and in vivo, but also safety, efficiency, and cost‐effectiveness. Progress in this area is expected to provide clear and effective insights for policy‐making and for decision‐making in the individualized treatment of EM.

## 3 POTENTIAL NEW PHARMACEUTICALS AND THEIR TARGET‐SIGNALING PATHWAYS

Medications investigated in ongoing or completed clinical trials on EM are summarized in Table 2. Most drugs are mainly symptomatic. Outcome measures used in these studies are pain score, levels of dysmenorrhea and dyspareunia, and quality of life, except for epigallocatechin gallate (EGCG) and quinagolide, whose efficacy in reducing endometriotic lesions will be determined. To the best of our knowledge, there is limited clinical trial to examine the pathophysiology or signaling pathways targeted by the drugs. Moreover, heterogeneous pathophysiology among patients affects their responsiveness to drug treatment; therefore, the development of personalized medicines to specific patients based on EM pathophysiology is desirable.^39^ These further emphasizes the demand for new pharmaceutical that is for symptomatic management, as well as targets specific pathophysiology and signaling pathways to eliminate the endometriotic lesions.

**Table 2:** Pharmaceuticals under clinical trials within 2015–2025 for endometriosis (EM) treatment^a^

NCT number	Study completion	Phase	Study locations or centers	Medication	Control	Study aim	Outcome measures^b^
NCT01769781	Jul‐15	Phase 4	Italy	Anastrazole plus GnRH‐agonist	GnRH analog	Efficacy of drug for EM recurrence	Pelvic pain
NCT01767090	Jul‐15	Phase 2	Belgium, Japan, United Kingdom, and so forth	ASP1707	Placebo and Leuprorelin acetate	Safety and efficacy of drug in different doses for EM‐associated symptoms	Pelvic pain, dysmenorrhea, dyspareunia, adverse events, bleeding pattern
NCT01779232	Sep‐15	Phase 4	Italy	Danazol	Placebo	Efficacy of drug for EM‐related infertility	Fertilization outcome
NCT01822080	Nov‐15	Phase 3	China	Dienogest	Placebo	Efficacy of drug for EM‐related symptoms in Chinese Patients	Pelvic pain, dysmenorrhea, adverse events, bleeding pattern
NCT02475564	Nov‐15	Phase 4	Brazil	Resveratrol	Placebo	Efficacy of drug for EM‐related pain	Pelvic pain, CA125, and prolactin marker
NCT01712763	Mar‐16	Phase 3	Italy	Degarelix	Goserelin	Efficacy of drug for EM recurrence	Pelvic pain
NCT01760954	Apr‐16	Phase 3	AbbVie Inc.	Elagolix	N/A	Long‐term safety and efficacy of drug for EM‐related symptoms	Pelvic pain, dysmenorrhea, dyspareunia, quality of life, adverse Events
NCT02534688	May‐16	Phase 4	Thailand	LNG‐IUS and DMPA	N/A	Efficacy of drug for EM‐related symptoms	Pelvic pain, quality of life, hormone profile
NCT02387931	Jul‐16	Phase 4	United States	Vitamin D3 and Fish Oil	Placebo	Efficacy of drug for adolescent girls with EM‐related symptoms	Pelvic pain, quality of life
NCT02427386	Dec‐16	Phase 4	University of Sao Paulo General Hospital	Dynamized estrogen	Placebo	Efficacy of drug for EM‐related symptoms	Pelvic pain
NCT01931670	Dec‐16	Phase 3	AbbVie Inc.	Elagolix	Placebo	Safety and efficacy of drug for EM‐related symptoms	Pelvic pain, dysmenorrhea, dyspareunia, quality of life
NCT01728454	Mar‐17	Phase 2	United States	Telapristone acetate	Placebo	Safety and efficacy of drug for EM‐related symptoms in premenopausal women	Pelvic pain, dysmenorrhea, dyspareunia
NCT02143713	May‐17	Phase 3	AbbVie Inc.	Elagolix	Placebo	Long‐term safety and efficacy of drug for EM‐related symptoms	Pelvic pain, dysmenorrhea, dyspareunia, quality of life, adverse Events
NCT02480647	Aug‐17	Phase 4	Brazil	Levonorgestrel and Etonogestrel	N/A	Efficacy of drug for EM‐related symptoms	Pelvic pain, bleeding pattern
NCT02542410	Sep‐18	Phase 2	United States	Cabergoline	Norethindrone acetate	Efficacy of drug for EM‐related symptoms	Pelvic pain
NCT02778399	Jul‐19	Phase 2	United States, Poland, Russian Federation, Ukraine, and so forth	OBE2109	Placebo	Efficacy and safety for EM‐related symptoms	Pelvic pain, dysmenorrhea, dyspareunia, dyschezia, quality of life, adverse events
NCT01553201	Jul‐19	Phase 1|Phase 2	United States	Botulinum Toxin	Placebo	Efficacy of drug for EM‐related symptoms	Pelvic pain
NCT03232281	Nov‐19	Phase 3	China	Triptorelin Pamoate PR 3‐month and Triptorelin Acetate PR 1‐month	N/A	Efficacy and safety of drug for EM‐related symptoms in Chinese patients	Pelvic pain, percentage of subjects castrated, hormones profile
NCT03340324	Dec‐19	Phase 2	Mongolia	V‐Endo	N/A	Efficacy of drug for EM‐related symptoms	Pelvic pain, complete blood count, liver and kidney function
NCT03352076	May‐20	Phase 2	Italy	Vaginal danazol and oral danatrol	N/A	Concentration of drug for EM‐related symptoms	Danazol concentration
NCT03654326	Jun‐20	Phase 2	United States, Australia, Chile, New Zealand, and so forth	Gefapixant	Naproxen and Placebo	Efficacy and safety of drug for EM‐related symptoms	Pelvic pain, adverse events
NCT03931915	Sep‐20	Phase 3	Japan	TAK‐385 and leuprorelin acetate	N/A	Efficacy and safety of drug for EM‐related symptoms	Pelvic pain, dyspareunia, adverse events, serum concentrations, menstrual pain
NCT03573336	Oct‐20	Phase 2	United States, Austria, Canada, Japan, and so forth	Vilaprisan	Placebo	Efficacy and safety of drug for EM‐related symptoms	Pelvic pain, adverse events, clinical imaging assessments
NCT02832271	Dec‐20	Phase 2	Hong Kong	SUNPHENON EGCG	Placebo	Efficacy and safety of drug for EM‐related symptoms and lesion size	Pelvic pain, lesion size, quality of life, adverse events
NCT03782740	Feb‐21	Phase 2	Sweden	Melatonin	Placebo	Efficacy and safety of drug for EM‐related symptoms	Pelvic pain, quality of life, adverse events, acceptance of melatonin
NCT03204331	Mar‐21	Phase 3	United States, Australia, Brazil, Chile, and so forth	Relugolix	Estradiol/norethindrone acetate, Placebo	Efficacy and safety of drug for EM‐related symptoms	Pelvic pain, dysmenorrhea, dyspareunia, quality of life, adverse events, hormone profiles
NCT03204318							
NCT03749109	May‐21	Phase 2	Denmark, Germany, Italy, Poland, and so forth	Quinagolide	Placebo	Efficacy and safety of drug for EM‐related symptoms and lesion size	Lesion size, dysmenorrhea, quality of life, adverse events, clinical imaging assessments
NCT01942122	Jun‐21	Phase 2|Phase 3	Indonesia	DLBS1442	Mefenamic acid	Efficacy of drug for EM‐related symptoms	Pelvic pain, quality of life, adverse events, inflammatory markers, hormone profile
NCT03840993	Aug‐21	Phase 2	United States	MT‐2990	Placebo	Efficacy and safety of drug for EM‐related symptoms	Pelvic pain
NCT04256200	Dec‐21	Phase 2|Phase 3	Lebanon	Dienogest 2‐mg oral tablet	Oral Contraceptive Pills	Efficacy and safety of drug for EM‐related symptoms	Pelvic pain, quality of life, adverse events, patient tolerability
NCT03991520	Jan‐22	Early Phase 1	United States	Anakinra 100‐mg/0.67‐ml Inj Syringe	Placebo	Efficacy for EM‐related symptoms (pilot study)	Pelvic pain, dysmenorrhea, dyspareunia, quality of life, serum inflammatory markers
NCT03992846	Jul‐22	Phase 3	United States, Austria, Bulgaria, Czechia, and so forth	Linzagolix	Placebo	Efficacy of drug for EM‐related symptoms	Pelvic pain, dysmenorrhea, dyspareunia, dyschezia
NCT03986944							
NCT03654274	Dec‐22	Phase 3	United States, Argentina, Australia, Belgium, and so forth	Relugolix	Estradiol/norethindrone acetate	Efficacy and safety of drug for EM‐related symptoms	Pelvic pain, dysmenorrhea, dyspareunia, adverse events, hormone profile
NCT03213457	Jan‐23	Phase 3	United States, Canada, Puerto Rico	Elagolix, estradiol/norethindrone acetate	Placebo	Efficacy and safety of drug for EM‐related symptoms	Pelvic pain, dysmenorrhea, dyspareunia
NCT03928288	Feb‐23	Phase 2	United States	Cabergoline	Placebo	Efficacy of drug for EM‐related symptoms	Pelvic pain, dysmenorrhea, serum angiogenesis and inflammatory biomarkers, adverse events
NCT03970330	May‐23	Phase 3	United States	Naltrexone and norethindrone acetate	Placebo	Efficacy of drug for EM‐related symptoms	Pelvic pain
NCT03692403	Aug‐23	Phase 2	United States	Quinagolide	Placebo	Efficacy of drug for EM‐related symptoms	Pelvic pain, dysmenorrhea, dyspareunia, quality of life, bleeding pattern, adverse events

^a^ All information was taken from the US National Library of Medicine, ClinicalTrials.gov, only completed or active clinical trials, and EM treatment as the primary study purpose between 2015 and 2025 are included.

^b^ Selected outcome measures are shown.

Here, we discuss the pathophysiology and molecular targets that are directly or indirectly associated with the drugs, as well as their effects on the corresponding signal transduction pathways in the treatment of EM. In Table 3, we distinguished potential drugs as a repurposed or a de novo drug of EM. A new drug is defined as a chemical that has not been studied in clinical trials for other diseases before EM and a repurposed drug is defined as a chemical that has been studied in clinical trials for other diseases before EM. We provided sufficient scientific data of their efficacies in reducing endometriotic cell viability in vitro or lesions in vivo, as well as in regulating specific signaling pathways and molecules involved in the pathophysiology of EM. The advantages, side effects, and limitations of the drugs are also highlighted.

**Table 3:** Pathways and molecular targets of current and potential pharmaceuticals for endometriosis treatment

Pathways	Molecular targets	Pathophysiology	Medication	PK and toxicity profile (accession number if available)^a^	Drug development approach^b^	Original purpose of drugs before EM (phase, start/stated year)^c^	Type/Class^d^	Clinical stages as EM treatment
Current medication								
COX‐2	COX‐1, COX‐2, PPAR‐γ	Proliferation	Indomethacin	PK/toxicity (DB00328)^h^	Repurpose	Anti‐inflammatory Agent (since 1963)	Nonhormone, NSAID	Preclinical, off‐label prescription
COX‐2/PGE_2_, COX‐2/VEGF	COX‐2	Proliferation and apoptosis	Celecoxib	PK/toxicity (DB00482)^h^	Repurpose	Arthritis (IV, 2002)	Nonhormone, NSAID	Preclinical, off‐label prescription
NF‐κB	TNF‐*α*	Proliferation and inflammation	GnRH agonist	PK/toxicity (DB11979, DB00050)^h^	Repurpose	Contraceptive agents (since 1978)	Hormone, GnRH agonist	Phase 4, on‐label prescription
TNF*α*/NF‐κB	NF‐κB	Proliferation and inflammation	Progesterone or dienogest or danazol	PK/toxicity (DB00396, DB09123, DB01406)^h^	Repurpose	NA	Hormones, progestogen & contraceptives	Phase 4, on‐label prescription
VEGF and IL‐8 mediated apoptosis	GnRH	Apoptosis and inflammation	Leuprolide acetate	PK/toxicity (DB00007)^h^	Repurpose	Prostate cancer (since 1985)	Hormone, GnRH agonist	Phase 4, on‐label prescription
PI3K/Akt/mTOR and MEK1/2/ERK1/2	AKT and ERK1/2	Apoptosis and autophagy	Dienogest	PK/toxicity (DB09123)^h^	Repurpose	Oral contraceptive (III, 2003)	Hormone, progestogen	Phase 3, on‐label prescription
Antiproliferation and proapoptotic agents								
CASP and apoptotic proteins effects	NF‐κB, IκB, JNK, p38 MAPK	Proliferation and apoptosis	BAY11‐7085	No information	New	NA	Nonhormones, NF‐κB inhibitor	Preclinical
CASP	MMP‐3	Apoptosis	Melatonin	PK/toxicity (DB01065)^h^	Repurpose	Insomnia (IV, 2007), chemotherapy‐induced toxicity (III, 2007), prevention of lung cancer (III, 2007)	Hormone, miscellaneous anxiolytics, sedatives, and hypnotics	Phase 2
E_2_/ER	ERβ	Proliferation, inflammation, angiogenesis, and apoptosis	Chloroindazole	No information	New	NA	Nonhormones, NA	Preclinical
E_2_/ER	ERα	Proliferation, inflammation, angiogenesis, and apoptosis	Oxabicycloheptene sulfonate	PK (DB04574)^h^	New	NA	Nonhormone, NA	Preclinical
E_2_/ER	ESR1	Proliferation	Resveratrol	PK/toxicity (drugs.com/resveratrol.)^i^	Repurpose	Inflammation in type 2 diabetic patients (III, 2013), Anti‐inflammatory and antioxidant effects (III, 2011)	Natural products, phytoalexin	Phase 4
E_2_/ER/VEGF	/	Proliferation and angiogenesis	EGCG	PK^67^/toxicity^68^	Repurpose	Multiple sclerosis (III, 2009), cervical cancer (II, 2005), prostate cancer (II, 2004)	Natural products, catechin	Phase 2
ER stress	TRAIL	Apoptosis	Tunicamycin	Toxicity^71^	New	NA	Nonhormones, antibiotic	Preclinical
Hypoxia/LATS1/YAP1	YAP1	Proliferation, angiogenesis, and migration	Verteporfin	PK/toxicity (DB00460)^h^	Repurpose	Neovascular macular degeneration (IV, 2014), polypoidal choroidal vasculopathy (IV, 2008)	Nonhormone, photosensitizing agent	Preclinical
NF‐κB	TNF‐*α*‐induced effect	Apoptosis and angiogenesis	Ginsenoside Rg3	PK^76^/Toxicity^77^	Repurpose	Endothelial Function (II, 2007)	Natural product, Steroid glycoside	Preclinical
p53/NF‐κB	MMP‐3	Apoptosis	Curcumin	PK/toxicity (DB11672)^h^	Repurpose	Inflammation in endometrial carcinoma (II, 2013), irritable bowel syndrome (IV, 2008)	Natural products, curcuminoid	Recruiting
NF‐κB and COX‐2	TGF‐β	Apoptosis	Genistein	PK^82^	Repurpose	Endothelial function (III, 2010), vascular and skeletal protective in menopause women (III, 2003), prostate cancer (III, 2003)	Natural product, isoflavone	Preclinical
RAF/MEK/ERK and VEGF/VEGFR	RAF and VEGFR	Proliferation, inflammation, and angiogenesis	Sorafenib	PK/toxicity (DB00398)^h^	Repurpose	Hepatocellular carcinoma (IV, 2010) carcinoma, renal cell (III, 2003)	Nonhormone, multikinase inhibitor	Preclinical
MAPK/ERK	BARF	Proliferation and apoptosis	Vemurafenib	PK/toxicity (DB08881)^h^	Repurpose	Malignant Melanoma (III, 2010)	Nonhormone, kinase inhibitor	Preclinical
MAPK/PR	MEK1/2	Proliferation and apoptosis	U0126	PK^90^	New	NA	Nonhormone, MAPK/ERK kinase	Preclinical
MAPK/ERK1/2	ERs	Proliferation	Puerarin	PK^92^	Repurpose	Alcohol abuse (II, 2009)	Natural product, isoflavonoid	Preclinical
EGFR/ERK1/2, AKT, B‐catenin, NF‐κB	EP2 and EP4 receptors	Apoptosis	PGE_2_ inhibitors	PK/toxicity (DB00917)^h^	Repurpose	Cataracts (IV, 2007)	Nonhormone, PGE2 inhibitors	Preclinical
mTOR/Akt	CB1 or CB2	Proliferation, fibrogenesis, and oxidation	WIN 55212‐2	PK^96^	New	NA	Nonhormone, cannabinoid receptor agonist	Preclinical
Akt/PR	AKT	Proliferation and apoptosis	MK2206	No information	Repurpose	Recurrent ovarian carcinoma (II, 2012), endometrial adenocarcinoma (II, 2012)	Nonhormone, AKT inhibitor	Preclinical
p53, p21, CASP, FOXO, inducing apoptosis	/	Proliferation and apoptosis	Propofol	PK/toxicity (DB00818)^h^	Repurpose	Anaesthesia (IV, 2001)	Nonhormone, aesthetic	Preclinical
Metabolic process	PDH kinase	Proliferation	Dichloroacetate	PK^101^/Toxicity (DB08809)^h^	Repurpose	Brain cancer (II, 2007), lactic acidosis (III, 1998)	Nonhormone, alpha‐halocarboxylic acid	Preclinical
Autophagy modulators								
ATG regulated autophagy	/	Autophagy, proliferation, and apoptosis	MK2206 and chloroquine	NA	NA	NA	Combination therapy; AKT inhibitor (MK2206) and amebicide (chloroquine	Preclinical
E_2_/ER and PR	ER*α* and PR*α*	Autophagy and inflammation	Ginsenoside PPD	PK^76^/Toxicity^77^	Repurpose	Endothelial Function (II, 2007)	Natural product, Steroid glycoside	Preclinical
ERK and Beclin1 inducing autophagy, CDK	Beclin‐1 and ERK autophagy‐promoting proteins, p27	Proliferation, apoptosis, and autophagy	MIS	No information	Repurpose	PCOS (III, 2012)	Hormone, glycoprotein hormone	Preclinical
Antimigration, anti‐invasion, and antifibrosis agents								
CBP/β‐catenin	CBP/β‐catenin complex	Proliferation, migration, apoptosis, and fibrogenesis	C‐82	PK^105^	Repurpose	Systemic scleroderma (II, 2015), psoriasis (II, 2015)	Nonhormone, β‐catenin inhibitor	Preclinical
CBP/β‐catenin	CBP/β‐catenin complex	Proliferation, migration, apoptosis, and fibrogenesis	ICG‐001	No information	Repurpose	Myeloid Leukaemia (II, 2012)	Nonhormone, β‐catenin inhibitor	Preclinical
Wnt/β ‐catenin	Tcf/β‐cateini complex	Proliferation, migration, and invasion	PKF115‐584	No information	New	NA	Natural product, NA	Preclinical
Wnt2/β‐catenin	/	Invasion	Metformin	PK/toxicity (DB00331)^h^	Repurpose	PCOS (IV, 2003), type 2 diabetes (IV, 2000)	Nonhormone, antidiabetics and biguanides	Preclinical
Wnt/β‐catenin	Tcf/β‐cateini complex	Proliferation, migration, invasion, and fibrogenesis	PKF115‐584/CGP049090	No information	New	NA	Natural product, NA	Preclinical
TGF‐β1‐stimulated activation of MAPK and Smad pathway	/	Proliferation, migration, invasion, and fibrogenesis	EGCG	PK^67^/toxicity^68^	Repurpose	Multiple sclerosis (III, 2009), cervical cancer (II, 2005), prostate cancer (II, 2004)	Natural product, catechin	Phase 2
NF‐κB/MMP‐2/MMP‐9	NF‐κB	Invasion	Genistein	PK^82^	Repurpose	Endothelial Function (III, 2010), vascular and skeletal protective in menopause women (III, 2003), prostate cancer (III, 2003)	Natural product, isoflavone	Preclinical
Rho/ROCK	ROCK	Proliferation, apoptosis, contractility, and differentiation	Fasudil	PK^113^	Repurpose	Raynaud, scleroderma (III, 2007)	Nonhormone, Rho‐kinase inhibitor, and vasodilator	Preclinical
Rho/ROCK	/	Fibrogenesis and differentiation	Heparin	PK/toxicity (DB01109)^h^	Repurpose	Thrombosis (IV, 1997), inflammation (IV, 2008), anticoagulation (IV, 2009), Cancer (IV, 2009), IVF‐ET failure, and thrombophilia (IV, 2009)	Nonhormone, anti‐inflammatory agent	Preclinical
Antiangiogenesis agents								
Multikinase	VEGFR, PDGFR	Apoptosis and angiogenesis	Sunitinib (SU11248)	PK/toxicity (DB01268)^h^	Repurpose	Carcinoma, renal cell (IV, 2008), gastrointestinal stromal tumors (IV, 2008)	Nonhormone, multikinase inhibitor	Preclinical
Multikinase	VEGFR‐2, FGFR‐1 and PDGFR‐β	Angiogenesis	SU6668	PK/toxicity^121^	Repurpose	Hepatocellular carcinoma (II, 2003)	Nonhormone, multikinase inhibitor	Preclinical
VEGF/VEGFR	VEGFR2	Angiogenesis	SU5416	PK/toxicity^124^	Repurpose	Melanoma (II, 2001), malignant mesothelioma (II, 2000), gastrointestinal stromal tumour, sarcoma (II, 2000)	Nonhormone, VEGFR inhibitor‎	Preclinical
VEGF/VEGFR	VEGF	Angiogenesis and proliferation	Pazopanib (P), sunitinib (SU) and sorafenib (SO)	(P) PK(DB06589)^h^, others as mentioned	(P) repurpose, others as mentioned	(P) Cancer (IV, 2010), ovarian cancer (III, 2009), carcinoma, renal cell (II, 2006) others as mentioned	(P) Nonhormone, multikinase inhibitor, others as mentioned	Preclinical
VEGFC mediated c‐JUN, IFN‐γ, CXCL3, and MMP‐9 pathway	VEGFC/VEGFR2	Proliferation and angiogenesis	EGCG	PK^67^/toxicity^68^	Repurpose	Multiple sclerosis (III, 2009), cervical cancer (II, 2005), prostate cancer (II, 2004)	Natural product, catechin	Phase 2
VEGF	VEGF	Proliferation, angiogenesis and oxidative stress	ProEGCG	No information,	New	NA	Natural product, prodrug	Preclinical
NF‐κB/TNF‐*α*/VEGF	NF‐κB	Angiogenesis	Pyrrolidine dithiocarbamate	PK/toxicity^131^	New	NA	Nonhormone, metal chelator	Preclinical
VEGFC and VEGFR2	VEGFC and VEGFR2	Angiogenesis	PTX	PK/toxicity (DB00806)^h^	Repurpose	Hemodialysis (IV, 2006), intermittent claudication (since 1982)	Nonhormone, hemorheological agent	Phase 3
VEGF/VEGFR2	Dopamine receptor 2	Angiogenesis and inflammatory	Quinagolide	PK/toxicity (DB09097)^h^	Repurpose	Hyperprolactinaemia (since 1994)^134^	Nonhormone, dopamine agonist	Phase 4
Antioxidative stress agents								
Radical scavenging activity/ERK	ROS	Oxidative stress and proliferation	NAC	PK/toxicity (DB06151)^h^	Repurpose	Cystic fibrosis (II, 2008), multiple sclerosis (II, 2004), pulmonary fibrosis (III, 2000),	Nonhormone, antidote	Preclinical
Regulation of antioxidant enzymes	ROS	Oxidative stress	Resveratrol	PK/toxicity (drugs.com/resveratrol.)^i^	Repurpose	Inflammation in type 2 diabetic patients (III, 2013), anti‐inflammatory and antioxidant effects (III, 2011)	Natural product, phytoalexin	Phase 4
Regulation of antioxidant enzymes	ROS	Oxidative stress	Caffeic Acid	PK^139^	Repurpose	Immune thrombocytopenia (III, 2012)	Natural product, phenolic acid	Preclinical
Radical scavenging activity	ROS, MMP, VEGF	Oxidative stress, angiogenesis, and inflammation	Melatonin	PK/toxicity (DB01065)	Repurpose	Insomnia (IV, 2007), chemotherapy‐induced toxicity (III, 2007), prevention of lung cancer (III, 2007)	Hormone, miscellaneous anxiolytics, sedatives, and hypnotics	Phase 2
Anti‐inflammation agents								
Cytokines	/	Proliferation, invasion and inflammation	NAC	PK/toxicity (DB06151)^h^	Repurpose	Pulmonary fibrosis (III, 2000), cystic fibrosis (II, 2008), multiple sclerosis (II, 2004)	Nonhormone, antidote	Preclinical
Cytokines	/	Proliferation and inflammation	Crocin	PK^145^/toxicity^146^	Repurpose	Metabolic syndrome (IV, 2010)	Natural product, diterpenoid	Preclinical
Cytokines	/	Inflammation	Metformin	PK/toxicity (DB00331)^h^	Repurpose	PCOS (IV, 2003), type 2 diabetes (IV, 2000)	Nonhormone, antidiabetics and biguanides	Preclinical
Cytokines	/	Angiogenesis and inflammation	Resveratrol	PK/toxicity (drugs.com/resveratrol.)^i^	Repurpose	Inflammation in type 2 diabetic patients (III, 2013), anti‐inflammatory and antioxidant effects (III, 2011)	Natural product, phytoalexin	Phase 4
TNF*α*‐mediated cytokines	SIRT1	Inflammation	Resveratrol	PK/toxicity (drugs.com/resveratrol.)	Repurpose	Inflammation in type 2 diabetic patients (III, 2013), anti‐inflammatory and antioxidant effects (III, 2011)	Natural product, phytoalexin	Phase 4
Cytokines	MIF	Angiogenesis and inflammation	ISO‐1	No information	New	NA	Nonhormone, MIF inhibitor	Preclinical
E_2_/ER	P450AROM	Inflammation	Puerarin	PK^92^	Repurpose	Alcohol abuse (II, 2009)	Natural product, isoflavonoid	Preclinical
MAPK, Wnt pathway	/	Proliferation, angiogenesis, and inflammation	Niclosamide	PK/toxicity (DB06803)^h^	Repurpose	Anthelmintic (since 1982)	Nonhormone, anthelmintic agent	Preclinical
IκKα/β, NF‐κB, STAT3, and JNK	Chemokine and cytokines	Angiogenesis and inflammation	Curcumin	PK/toxicity (DB11672)^h^	Repurpose	Inflammation in endometrial carcinoma (II, 2013), irritable bowel syndrome (IV, 2008)	Natural product, curcuminoid	Recruiting
NK cells cytotoxicity	/	Immune system	Ginsenoside PPD	PK^76^/Toxicity^1^, ^77^	Repurpose	Endothelial function (II, 2007)	Natural product, steroid glycoside	Preclinical
VEGF/VEGFR, iNOS/NO, COX‐2/PGE_2_	VEGF, iNOS, and COX‐2	Angiogenesis and inflammation	Acai	PK/toxicity (drugs.com/acai)^i^	Repurpose	Antioxidant (2010, III), prostate cancer (2011, II)	Natural product, anthocyanin	Preclinical

Medication	Study models	Positive control group	Negative control group	Assessments	Efficacy (compared to untreated)^e^, ^f^, ^g^	Size effects or other comments	Reference
Current medication							
Indomethacin	Animals (EM mice model)	NA	Vehicle	Lesions assessment	↓~46% in area of all lesions	Stomach upset, headache, drowsiness, dizziness, and so forth.^1^	[60]
Celecoxib	Cells (Primary human endometriotic stromal cells)	NA	Vehicle	Proliferation and apoptosis assays, IHC, Western blot, ELISA	↓~60% in proliferation, ~50% VEGF and ~70% PGE_2_, ↑ ~3.25‐fold in apoptosis and ~2‐fold COX‐2 expression with 100 µM of celecoxib	Stomach upset, headache, drowsiness, dizziness, and so forth.^1^	[53]
GnRH agonist	Cells (primary human endometriotic stromal cells)	NA	Untreated	Western blot, EMA	↓~80% TNF‐*α* mediated IL‐8 level	Hypoestrogenic^31^, ^32^	[31]
Progesterone or dienogest or danazol	Cells (primary human endometriotic stromal cells)	NA	Untreated	EMA, ELISA, Northern blot analysis	↓~40% in TNF‐*α* mediated IL‐8 level	Hypoestrogenic^31^, ^32^	[50]
Leuprolide acetate	Cells (primary human eutopic epithelial endometriotic cells)	NA	Basal conditions	Apoptosis assay, ELISA	↑1.74‐fold in apoptosis level, ↓62.5% in IL‐8 level, and ↓52.6% in VEGF level	Hypoestrogenic^31^, ^32^	[52]
Dienogest	Cells (primary human endometriotic stromal cells)	AKT inhibitor VIII and U0126	Untreated	Western blot, TEM, IF, autophagy, and apoptosis assays	↑~1.5‐fold of LC3‐II and SQSTM1 expression, ~25% in autophagy level, ↓~40% in p‐Akt and p‐ERK	Hypoestrogenic^31^, ^32^	[61]
Antiproliferation and proapoptotic agents							
BAY11‐7085	Cells (primary human endometriotic and endometrial stromal cells)	NA	Untreated	MTT, ELISA, apoptosis assay, flow cytometry, Western blot	↓66.1% cell viability and ↑725.1% in apoptosis ability with 10 µM of BAY11‐7085 in ECSCs	No information	[62]
Melatonin	Animals (EM rat model)	Vehicle	NA	H&E, Western blot, RT‐PCR, EMSA, Tunel assay	↓~80% secreted proMMP‐3 and ↓ ~80% synthesized proMMP‐3 on 35th day	No side effects reported	[63]
Chloroindazole	Cells (primary human endometriotic stromal cell) and animals (EM mice model)	NA	Vehicle	Lesions assessment, WST‐1 assay, Tunel assay, qRT‐PCR, LC‐MS	↓~88% in lesions weight, ↓~90% in Ki67 and p65 cells, ↓~88% in IL‐6 cells, in the therapeutic model, ↓~60% cell viability	No adverse effects on the reproductive tract or disturb estrous cycling or fertility	[64]
Oxabicycloheptene sulfonate	Cells (primary human endometriotic stromal cell) and animals (EM mice model)	NA	Vehicle	Lesions assessment, WST‐1 assay, Tunel assay, qRT‐PCR, LC‐MS	↓~78% in lesions weight, ↓~85% in Ki67 and p65 cells, ↓~78% in IL‐6 cells, in the therapeutic model, ↓~60% cell viability	No adverse effects on the reproductive tract or disturb estrous cycling or fertility	[64]
Resveratrol	Cell line (Ishikawa cells) and animals (EM xenograft model)	Progesterone	Vehicle	Alkaline phosphatase assay, IHC, RT‐PCR	↓~50% ESR1 and ~60% Ki67 expression in epithelium in high dose	Mild, mainly related to headache and somnolence^65^	[66]
EGCG	Cells (primary human endometrial stromal and glandular cells), and animals (EM Syrian golden hamsters model)	NA	DMSO in vehicle (animal) and vehicles (cells)	Lesions and microvessel assessment, WST‐1 assay, Western blot, Intravital fluorescence microscopy, H&E	↓~28.5% of E_2_ induced activation and ~33.3% E_2_ induced VEGF in EGC; ↓~38.5% endometriotic lesions regression; ↓50% of volumetric blood flow in endometriotic lesions on Day 14	Well tolerated, only mild headache and fatigue^69^	[70]
Tunicamycin	Cells (primary human endometriotic and endometrial stromal cells)	TRAIL	Vehicle	qRT‐PCR, Flow cytometry	↑59.1% in apoptosis (−TRAIL) ↑1.35‐fold in apoptosis (+TRAIL) in women with EM	Major neurotoxicity and death in animals^72^	[73]
Verteporfin	Cells (primary human endometriotic stromal cells) and animals (EM mice model)	NA	Vehicle	Western blot, IHC, ChIP assay, MTS assay, GSEA, Lesions assessment,	↓Proliferation in a dose‐dependent manner, ↓~50% in migration and tube formation, ↓~57% in lesions weight	Visual disturbances^74^	[75]
Ginsenoside Rg3	Cells (primary human endometriotic stromal cells)	NA	Untreated	CCK8, Western blot, RT‐PCR	↓~40% cells after 72 h with 150 µg/ml Rg3, ↓ TNF‐*α* induced effect of NF‐κB p65 (~20%), VEGF (~25%) and ↑~25% TNF*α* induced effect of CASP3	No side effect reported from RCTs^78^	[79]
Curcumin	Animals (EM mice model)	Celecoxib	PBS	Lesions assessment, H&E, Western blot, AFM, electrophoresis	↓~80% lesions glands, ↓~60% of p65/NF‐κB expression, ↑~6‐fold of Bax/bcl2 ratio on Day15	Safe and well‐tolerated even at high dose^80^	[81]
Genistein	Animals (EM mice model)	Dienogest and Leuprolide acetate	Untreated	Lesions assessment, IHC	↓4% of TGF‐β and 4.5% NF‐κB, 3.4% Bcl‐2, 1.35% COX2, 2% PGE, ↑1.35% Bax expression levels with 1.30 mg/day of Genistein	No side effect reported^83^, ^84^	[85]
Sorafenib	Cells (primary Human endometriotic stromal cell), and animals (EM mice xenograft model)	NA	Untreated (cell) or placebo (animal)	Lesions assessment, crystal violet assay, Western Blot, H&E	↓99.7% decreased in ectopic stromal cell, ↓64% in pERK–ERK ratio, ~33.3% in pVEGFR‐VEGFR ratio, ~35% implants size	Weight loss, skin, and gastrointestinal toxicities^86^	[87]
Vemurafenib	Cells (primary human stromal/epithelial; endometriotic/endometrial cells) and animals (EM mice xenograft model)	NA	vehicle (1% DMSO in media)	Western blot, IHC, lesions assessment, viability, apoptosis, and crystal violet assay assays	↓viability (69%, 66.7%), ↓optic density of pERK/ERK (62%, 61%), BRAF/B‐actin (61%, 66%) in stromal, epithelial cells, ↓ 37% implants size	Arthralgias, rash, and hyperkeratosis^88^	[89]
U0126	Cells (primary human endometriotic and endometrial stromal cells)	Progestin (R5020)	DMSO	Immunoblotting, qRT‐PCR, Viability assay, IHC	↑~10% PRAB, ↓~20% viability in OSIS with 10uM of U0126	No information	[91]
Puerarin	Cells (primary human endometriotic stromal cells)	U0126	Vehicle	Binding assay, Western Blot, CCK‐8, qRT‐PCR, and phosphate kinase arrays	Relative binding affinity of 32.2% of ERs and puerarin complex, ↓~30% proliferation, and ERK related proteins: ↓~46% cyclin D1, ↓~73% COX‐2, and ↓~46% cyp19	Can be used for long periods without severe side effects	[93]
PGE_2_ inhibitors	Cell line (12Z and 22B), Cells (primary human endometriotic and endometrial stromal cells)	NA	Untreated	Western blot, IP, IF, TUNEL, RT‐PCR, IHC	↑Apoptosis in 12Z (~8‐fold) and 22B (~7‐fold), ↓ p‐EGFR/EGFR level in 12Z (~85%) and 22B (~63%), ↓ pERK/ERK level in 12Z (~50%) and 22B (~15%), ↓pAkt/Akt level in 12Z (~85%) and 22B (~72%), ↓B‐cate/B‐actin level in 12Z (~67%) and 22B (~43%),	GI upset, edema, and skin rash^94^	[95]
WIN 55212‐2	Cells (primary human stromal/epithelial; endometriotic/endometrial cells) and animals (EM mice xenograft model)	NA	PBS	Proliferation and viability assays, Western blot, lesions assessment	↓65% Akt level in endometriotic stromal cell and 50% that in endometrial stromal cell, ↓43% in lesions volume	Dizziness, drowsiness, sedation, dry mouth and cognitive impairment^97^	[98]
MK2206	Cells (primary human endometriotic and endometrial stromal cells) and animals (EM xenograft mice model)	Progestin (R5020)	DMSO in vehicle (cell) and PBS (animal)	Lesions assessment, Western blot, qRT‐PCR, viability assay, IHC	↑~20% PRA and 30% PRAB, ↓~30% viability in OSIS with 10 µM of MK2206, ↓ ~20% tumor volume	No information	[91]
Propofol	Cell line (CRL‐7566)	NA	0.2% DMSO in vehicle	MTT, Flow cytometry, RT‐qPCR, Western blot	↑~35% in apoptosis level with 10 µg/ml propofol, ↑ FOXO1, FOXO3, Bim, p21, p53, CASP3 expression levels in a dose‐dependent manner	Hemodynamic instability, pain on injection, dystonic movements, hypertriglyceridemia, pancreatitis, allergic reactions^99^	[100]
Dichloroacetate	Cells (primary human peritoneal mesothelial cells), cell line (SHT290), animals (EM mice model)	NA	Vehicle (water)	ECAR and OCR measurement, Enzymatic colorimetric kit, Lesions assessment	↓~40% TGF‐β1–stimulated HPMC lactate secretion, ~25% cell proliferation, ↑ ~3‐fold PDH activity, ↓~20% lactate concentrations in PF, and ~50% lesions size	Peripheral neuropathy	[46]
Autophagy modulators							
MK2206 and chloroquine	Cells (primary human stromal/epithelial; endometriotic/endometrial cells) and animals (EM mice xenograft model)	NA	Vehicle	MTS, flow cytometry, transfection, Western Blot, IF, clonogenic assay, IHC	↓>80% proliferation in both cell growth and regrowth model in all cells and, >90% colony formation in both DES and EES, ↓>80% lesions volume, ↑>50% apoptosis in mice	Chloroquine alone might result in gastrointestinal symptoms but no information of adverse effects with combination treatment	[102]
Ginsenoside PPD	Cells (primary human endometriotic and endometrial stromal cells) and animals (EM mice model)	Esculentoside A	0.1% DMSO in vehicle	CCK8, flow cytometry, RT^2^ profiler™ PCR, Western blot, IHC, IF	>3‐fold difference in autophagy‐related genes ATG2, ATG3, and ATG5, ESR1, SQSTM1, and TGF‐B1, ↓~90% lesions weight and ~85% of lesions numbers with high dose PPD	Safe, low doses had no influence on growth of nESCs or the eutopic endometrium	[103]
MIS	Cell line (CRL‐7566)	NA	PBS	MTT, FACS analysis, flow cytometric analysis, Western blot	↓50% viability, ↑1.8‐fold proptosis with 10 µg/ml MIS	No information	[104]
Antimigration, anti‐invasion, and antifibrosis agents							
C‐82	Cells (primary human endometriotic and endometrial stromal cells)	NA	Vehicle	IHC, MTT, Western blot, ELISA, qRT‐PCR, scratch assay	↓51.8% cell viability, 91.9% cell proliferation, ↑200% apoptosis and 234.2% CASP3/7, ↓54% cell migration, all with 20 µM of C‐82	Mild, e.g reaction at the injection site, nausea, and constipation	[106]
ICG‐001	Cells (primary human endometriotic and endometrial stromal cells) and animals (EM mice model)	NA	Vehicle	IHC, MTT, Western blot, ELISA, qRT‐PCR, scratch assay, lesions assessment	↓20.8% cell viability, 86.1% cell proliferation, ↑56.4% apoptosis, 128.9% CASP3/7, ↓64%cell migration, all with 20 µM of ICG‐001, ↓~87.5% number of lesions	High dose was required for preclinical study and might not be comparable to clinical study	[106]
PKF115‐584	Cells (primary human stromal/epithelial; endometriotic/endometrial cells)	NA	Vehicle	qRT‐PCR, proliferation and migration assay, Western blot	↓73% invasion in endometriotic epithelial cells and 75% invasion, 85% MMP‐9 in stromal cells	Wnt/B‐catenin is needed for stem cell maintenance and tissue homeostasis^107^	[108]
Metformin	Cells (primary human endometriotic and endometrial stromal cells, endometrial epithelial cell)	NA	Untreated	MTT, Western blot	↓36.9% growth effects on epithelial cells by stromal factors, ↓~50% Wnt2 expression and secretion	Nausea and vomiting, diarrhea, abdominal pain^109^	[48]
PKF115‐584/CGP049090	Cells (primary human endometriotic and endometrial stromal cells)	NA	Vehicle	qRT‐PCR, MTS, migration assay, ICC, collagen gel contraction assay	↓>70% mRNA levels of *α*SMA, COL‐I, FN, CTGF in endometriotic stromal cell, collagen gel contraction	Wnt/B‐catenin is needed for stem cell maintenance and tissue homeostasis^107^	[110, 111]
EGCG	Cells (primary human endometriotic and endometrial stromal cells) and animals (EM mice model)	NAC	Vehicle	qRT‐PCR, MTS, migration and invasion assays, Collagen gel contraction assay, ICC, Western blot, H&E	↓>90% mRNA levels of *α*SMA, COL‐I, FN, CTGF in endometriotic stromal cell, ↓ > 90% migrated and invasive cells	Well tolerated, only mild headache and fatigue^69^	[110]
Genistein	Animals (EM mice model) and In silico	NA	Untreated	Lesions assessment, ELISA, docking	↑21.91 kJ/mol and 63.14 kJ/mol of NF‐κB to MMP‐2/‐9 binding energy, ↓~50% MMP‐2, ~30% MMP‐9 expression level with 100 mg/day genistein	No side effect reported^83^, ^84^	[112]
Fasudil	Cells (Primary human endometriotic stromal cells)	NA	Untreated	Cell viability assay, ELISA, flow cytometry, Collagen gel contraction assay, Western blot, Morphology assessment	↓43.7% proliferation, ~50% cell density, ~25% Bcl‐2, ~50% Bcl‐xl, *α*SMA, ROCKI, and ROCKII protein expressions, ~↑2‐fold apoptosis, 50% contractility, all with 100 µM Fasudil	Systemic vasodilation and hypotension^114^	[115]
Heparin	Cells (Primary human endometriotic stromal cells)	NA	Untreated	Morphology assessment, collagen gel contraction assay, Western blot	↓55.7% gel contraction with 100 mg/ml heparin sodium solution, ↓ *α*SMA, RhoA, ROCKI, and ROCKII expressions levels	Thrombocytopenia^116^	[117]
Antiangiogenesis agents							
Sunitinib (SU11248)	Animals (EM rat model)	NA	Saline	Lesions assessment, H&E, and Tunel assay	↓78.8% in cross‐sectional area of the cyst, and 50% complete cyst disappearance in animal	fatigue, diarrhea, skin discoloration, and nausea^118^	[119, 120]
SU6668	Animals (EM golden hamster model)	NA	DMSO	Lesions assessment, H&E, IHC	↓~30% vascularized area of endometrial grafts, ↓~50% microvessel density, ↓~25% size of endometrial grafts	Fatigue, nausea, vomiting, diarrhea, pain, flu‐like complaints, anorexia, change of taste^122^	[123]
SU5416	Animals (EM golden hamster model)	NA	DMSO	Lesions assessment, H&E, IHC	↓~20% microvessel density, ↓~5% size of endometrial grafts	Headache, pain at injection site^125^	[123, 126]
Pazopanib (P), sunitinib (SU) and sorafenib (SO)	Animals (EM rat model)	NA	Saline	Lesions assessment, H&E, IHC	↓~16% (SU), ~45% (P) in EM score; ↓~83%(SO), ~66% (SU), ~50% (P) in VEGF; ↓~56% (SU), ~40% (P) in CD117	Adverse effects on reproductive functions in animal models, including ovulation inhibition, embryotoxicity^127^	[128]
EGCG	Cells (human microvascular endothelial cells), and animals (EM mice xenograft model)	Vitamin E	Saline	Lesions and microvessel imaging, microarray, qRT‐PCR, Western blot, IHC	↓85% size and total vessel area of lesions with human endometrium tissue, ↓48% VEGFC mRNA, ↓~50% VEGFC expression level	Well tolerated, only mild headache and fatigue^69^	[70, 129]
ProEGCG	Animals (EM mice xenograft model)	Vitamin E	PBS	Lesions and Microvessel imaging, H&E, Tunel assay, ELISA, ORAC assay	↓>90% in lesions weight and size, ↓>70% in VEGF concentration, and ↑>4‐fold ORAC capacity in plasma and lesions during intervention	No sign of side effects	[130]
Pyrrolidine dithiocarbamate	Cells (primary human endometriotic and endometrial stromal cells)	NA	NA	EMSA, RT‐PCR, and Western blot	↓63.5% in TNF*α*–induced NF‐κB‐DNA binding activity, 53.9% CD44s, 55.2% MMP‐9, 68.5% VEGF expression in endometriotic stromal cells with 50 umol/L PD	No information	[132]
PTX	Animals (EM Wistar rats model)	NA	0.9% NaCl in vehicle	Lesions assessment, H&E, IHC	↓37.9% mean implant volume per animal, ↓47% VEGFC, and 40.1% Flk‐1 in glandular cells	No sign of side effects	[133]
Quinagolide	Human (hyperprolactinemic patients with EM)	NA	NA	Lesions morphology assessment, IHC, IF, and superarray	↓69.5% lesions size and 35% of lesions vanished completely, ↓VEGF (5.8‐fold), CCL2 (6.1‐fold), ↑CCL10 (6.8‐fold)	Dizziness, nausea, and vomiting	[135]
Antioxidative stress agents							
NAC	Cells (primary human stromal/epithelial; endometriotic/endometrial cells), animals (EM mice xenograft model)	Danazol and Mifepristone	PBS or sesame oil	UV spectroscopy, viability assays, H&E, Western blot, antioxidant enzyme activities	↓40%–60% H_2_O_2_ concentrations and 30%–60% proliferation in mice, ↓88% ratio pERK/ERK in human OSIS	High amount cause nausea, vomiting^136^	[137]
Resveratrol	Animals (EM rat model)	NA	Vehicle	Lesions assessment, H&E, PCNA‐IHC, antioxidant enzyme activities	↓41.5% of implants, ↑~50% (serum) and ↑ ~2‐fold (tissues) in SOD and GSH‐px activities, ↓46% (serum) and 77% (tissue) MDA, all with high dose resveratrol	Mild, mainly related to headache and somnolence^65^	[138]
Caffeic Acid	Cells (primary human endometriotic and endometrial stromal cells)	NA	Untreated	IHC, MTT, antioxidant activity assays	↓48.8% MDA ↑90.7% GSH, 56% CAT, 81% GPx, 59.5% GR	Excessive consumption can have insomnia^140^	[141]
Melatonin	Animals (EM rat model)	Vehicle	NA	Antioxidant activity assays, H&E, IHC	↓68.1% weight of implants, ↓ 66.8% in volume weight and ↓ 75.2% histologic score, ↓ 24% MDA, ↓45.6% CAT and ↑ 1.77‐fold SOD activities level, ↓ 61% VEGF ↓ 70% MMP‐9	No negative effect on fertility	[142, 143]
Anti‐inflammation agents							
NAC	Animals (EM mice model)	NA	Vehicle	Lesions assessment, IHC, qRT‐PCR	↓60% lesions, ↓54% Ki67, ~50% COX2, >60% MMP‐9 levels	High amount cause nausea, vomiting^136^	[144]
Crocin	Animals (EM mice model), and cell line (HUVEC and THP‐1)	NA	Untreated and saline	Lesions assessment, IHC, qRT‐PCR, ELISA, FC	↓~44% lesion size and ~64% of lesion weight, ~80% of VEGF and ~60% PCNA mRNA levels, ↓>50% of INF‐γ, TNF‐α, VEGF, and IL‐6 in serum	Mild effects like headache, insomnia, nausea, and dyspnea^147^	[148]
Metformin	Human (Stage1–2 EM patients)	NA	Placebo	ELISA	↓~35% IL6, ~33% IL‐8 and ~38% VEGF	Nausea and vomiting, diarrhea, abdominal pain^109^	[149]
Resveratrol	Animals (EM rat model)	NA	Vehicle	Lesions assessment, ELIZA, H&E, IHC	↓84.8% of implants volume, ↓VEGF level in Peritoneal fluid (54.8%), plasma (55.5%) and lesions (80.5%), ↓MCP‐1 in peritoneal fluid (48.3%)	Mild, mainly related to headache and somnolence^65^	[150]
Resveratrol	Cells (primary human endometriotic and endometrial stromal cells)	NA	Untreated	qRT‐PCR, CCK‐8, IHC, ELIZA	↓>60% TNF*α* induced IL‐8 mRNA expression and concentrations in ESC	Mild, mainly related to headache and somnolence^65^	[151]
ISO‐1	Animals (EM mice model)	NA	5% DMSO in vehicle	Lesions assessment, qRT‐PCR, Western blot	↓~70% in implant size, ~60% Flk‐1 expression, ~50% MIF activity	No information	[151]
Puerarin	Animals (EM rat model)	Raloxifene hydrochloride	Sodium carboxymethyl cellulose	Lesions and blood assessment, RT‐PCR, IHC	↓35.3% weight, ↓21.6% E2, 69.4% P450AROM, 41.5% COX‐2, 59.2%, 17*β*‐hsd‐1m, and ↑2.3‐fold 17*β*‐hsd‐2m mRNA of ectopic endometrium with M‐SI group	Can be used for long periods without severe side effects^93^	[152]
Niclosamide	Animals (EM mice model)	NA	Vehicle	Lesions assessment, IHC, IF, TUNEL, qPCR, GSEA	↓63.6% implant weight, 78.1% growth, 38.8% epithelial cell proliferation with 200 mg/kg niclosamid, ↓MAPK, Wnt, inflammation signaling‐related genes mRNA expression levels	Mild nausea and abdominal pain^153^	[154]
Curcumin	Cells (primary human endometriotic and endometrial stromal cells)	NA	0.1% DMSO in vehicle	Morphology assessment, Western blot, magnetic bead‐based assays	↑apoptosis, ↓phosphorylated form of IKKα/β, NF‐κB, STAT3, and JNK signaling, ↓10–15‐fold IL6, IL‐8, IP10, G‐CSF, MCP‐1 and RANTES, ↑ IL10, IL12, all in a dose‐dependent manner	Safe and well‐tolerated even at high dose^80^	[155]
Ginsenoside PPD	Cells (primary human endometriotic and endometrial stromal cells) and Animals (EM mice model)	Esculentoside A	0.1% DMSO in vehicle	CCK8, Flow cytometry, RT^2^ profiler™ PCR, Western blot, IHC, IF	↓~33.3% Bcl‐2, ~15% Bcl‐xL, and ~17% Ki‐67, ↑~28% CD82 in NK cells, ↓~67% E_2_ induced lesion, and ~70% E_2_ induced lesions weight	Safe, low doses had no influence on growth of nESCs or the eutopic endometrium	[103]
Acai	Cell line (J774.G8) and animals (Sprague‐Dawley rats)	NA	Vehicle	Lesions assessment, H&E, IHC, RT‐qPCR, ELIZA, flow cytometry, MTT	↓~92% lesions, ~33.3% VEGF conc., ~80% MMP‐9, 57% PGE2, ~50% viability with 40 µg/ml acai for 72 h	No information	[156]

Abbreviations: 17β‐hsd, 17β‐hydroxysteroid dehydrogenase; ‐SMA, ‐smooth muscle actin; AFM, atomic force microscopy; AKT, protein kinase B; P450AROM, aromatase; ASK1, apoptosis signal‐regulating kinase 1; ATF4, activating transcription factor 4; ATG, autophagy‐related protein; BRAF, serine/threonine‐protein kinase B‐Raf; CASP, caspases; CAT, catalase; CB, cannabinoid receptor; CCK8, cell counting kit‐8; CDK, cyclin‐dependent kinases; CHOP, CCAAT/enhancer‐binding protein homologous 10 protein; COL, collagen; COX, cyclooxygenase; CTGF, connective tissue growth factor; CXCL3, chemokine ligand 3; CYPs, cytochromes P450; DMSO, dimethyl sulfoxide; DVT, deep vein thrombosis; E_2_, estrogen; ECAR, extracellular acidification rate; EGCG, epigallocatechin gallate; EGFR, epidermal growth factor receptor; eIF2α, eukaryotic initiation factor 2 alpha; ELISA, enzyme‐linked immunosorbent assay; EM, endometriosis; ECSCs, endometriotic cyst stromal cells; EMSA, electrophoretic mobility shift assay; ER, estrogen receptor; ER stress, endoplasmic reticulum stress; ERK, extracellular signal‐regulated kinase; ESR1, estrogen receptor 1; FGFR, fibroblast growth factor receptors; Flk‐1, vascular endothelial growth factor receptor 2; FN, fibronectin; GnRH, gonadotropin‐releasing hormone; GI, gastrointestinal; GSHPx, glutathione peroxidase; GR, glutathione reductase; GRP, G protein‐coupled receptor; GSH, glutathione; H&E, haemotoxylin and eosin; ICC, immunocytochemistry; IF, immunofluorescence; IHC, immunohistochemistry; IFN‐γ, interferon‐γ; IL, interleukin; iNOS, inducible nitric oxide synthase; IRE1, inositol‐requiring enzyme 1; IκB, stimulate inhibitor of NF‐κB; IκK, IκB kinase; JNK, c‐Jun N‐terminal kinase; LATS1, large tumor suppressor kinase 1; LC, lapidated microtubule‐associated proteins 1 A/1B light chain; MAPK, mitogen‐activated protein kinase; MDA, malondialdehyde; MEK, ERK kinase; MIF, macrophage migration inhibitory factor; MIS, Mullerian‐inhibiting substance; MMP, matrix metallopeptidases; mTOR, mammalian target of rapamycin; mRNA, messenger RNA; MTT/MTS, cell proliferation assay; NAC, N‐acetyl cysteine; nESCs, normal endometrial stromal cells; NF‐κB, nuclear factor κB; NK cells, natural killer cells; NO, nitrogen oxide; OCR, oxygen consumption rate; ORAC, oxygen radical absorbance capacity; OSIS, endometriotic stromal cells; PCNA, proliferating cell nuclear antigen; PDGF, platelet‐derived growth factor; PDGFR, platelet‐derived growth factor receptor; PDH, pyruvate dehydrogenase kinase; PERK, endoplasmic reticulum kinase; PGE2, prostaglandin E2; PI3K, phosphoinositide 3‐kinases; PK, pharmacokinetics; PPAR, peroxisome proliferator‐activated receptor; PPD, protopanaxadiol; PR, progesterone receptor; ProEGCG, prodrug of EGCG; PTX, pentoxifylline; RAF, RAF proto‐oncogene serine/threonine‐protein kinase; RT‐qPCR, real‐time reverse‐transcription polymerase chain reaction; Rho, Ras homolog family; ROCK, Rho‐associated coiled‐coil kinase; ROS, reactive oxidative stress; SIRT1, sirtuin 1; SOD, superoxide dismutase; STAT, signal transducer and activator of transcription; SQSTM1, sequestosome 1; TCF, T‐cell factor; TCM, traditional Chinese medicine; TGF, transforming growth factors; TNF, tumor necrosis factor; TRAIL, TNF‐related apoptosis inducing ligand; TRAF2, TNF receptor‐associated factor 2; VEGF, vascular endothelial growth factor; VEGFR, vascular endothelial growth factor receptor; Wnt, wingless‐type mouse mammary tumor virus integration site family; WST‐1, cell proliferation assay; YAP, Hippo/Yes‐associated protein.

^a^ Pk and toxicity profile of drugs can be found on The Drugs.com Database, drugs.com, or on DrugBank Online, go.drugbank.com, or otherwise as stated.

^b^ A new drug is defined as a chemical that has not been studied in clinical trials for other diseases before EM and a repurposed drug is defined as a chemical that has been studied in clinical trials for other diseases before EM.

^c^ Representative clinical indications of drugs shows the original purpose before it was studied on EM. Information was taken from US National Library of Medicine, ClinicalTrials.gov, or otherwise as stated.

^d^ Data were extracted from The Drugs.com Database, drugs.com or DrugBank Online, go.drugbank.com.

^e^ Representative parameters were selected to show efficacy of drugs under corresponding pathophysiology.

^f^ Parameters of treated groups with a statistical difference of p < 0.05, compared to controls groups.

^g^ Data were extracted from tables or read from graphs.

^h^ Drug accession number is the ID of each drug entry on Drug bank.

^i^ Drug entry on the drug.com can be accessed via the URL.

### 3.1 Antiproliferative mechanism

#### 3.1.1 E_2_‐mediated pathway

Increased levels of E_2_ reduce progesterone and inhibit endoplasmic reticulum stress in endometrial cells.^157^ Increased expression of estrogen receptor (ER) isoforms has been observed in endometriotic lesions,^158^, ^159^ suggesting their contribution in regulating proliferation of the lesions. E_2_ is mediated by ERα and ERβ as well as by G protein‐coupled receptor 30 (GRP30), which is a seven‐transmembrane receptor. It activates phosphoinositide 3‐kinases (PI3K) and mitogen‐activated protein kinase (MAPK) through the transactivation of the epidermal growth factor receptor (EGFR) in the plasma membrane.^160^, ^161^ Chloroindazole and oxabicycloheptene sulfonate are two new chemicals bound to ERα and ERβ, respectively, and both inhibited E_2_‐driven proliferative and inflammatory activities in a dual action manner in ectopic lesions. This experimental study demonstrated great potential owing to their high potency and efficacy as preventive and therapeutic treatments. In addition, they do not exert any undesirable effects on the reproductive system. Co‐treatment of either ligands with letrozole enhanced the regression of ectopic endometrium, but it did not affect eutopic uterine tissues as with only letrozole.^64^ Increased COX‐2 and aromatase (P450AROM) levels stimulate E_2_ synthesis.^162^, ^163^, ^164^ P450AROM inhibitor maintained a low E_2_ level and reduced EM lesion size.^57^ COX‐2‐targeted treatment with celecoxib and indomethacin, which are two available NSAIDs, showed multiple effects on EM.^60^, ^165^ The drugs inhibited COX‐2‐mediated prostaglandin E_2_ (PGE_2_), which regulates E_2_ formation,^53^ but caused side effects including reproductive failures and cardiac adverse conditions.^166^

EGCG and resveratrol are both natural products that have been studied for EM treatment in clinical trials. They act as anti‐E_2_ agents, but with reduced side effects compared with hormonal drugs.^65^, ^69^ High doses of resveratrol reduced proliferation by interacting with ERα, and its expression in the endometrium epithelium was reduced to a profound level similar to that achieved with progesterone treatment. Nevertheless, progestogen did not reduce Ki‐67 expression in the endometrium stroma, whereas resveratrol reduced its expression in both the epithelium and stroma.^66^ EGCG inhibited E_2_‐stimulated proliferation and VEGF expression in cultured endometriotic glandular cells as well as angiogenesis and lesion growth via VEGF in mouse models.^70^, ^129^, ^167^

#### 3.1.2 NF‐κB pathway

NF‐κB is a protein that promotes cell proliferation and inhibits apoptosis in endometrial and endometriotic cells.^168^, ^169^, ^170^, ^171^ It is activated by cytokines, including TNF‐α, interleukins (IL)‐1β, and lipopolysaccharide. These stimulate inhibitors of NF‐κB (IκB) to be phosphorylated by IκB kinase (IκK).^171^ NF‐κB binds to DNA and transcripts the genes of angiogenic and adhesion factors, cytokines, growth factors, and inducible enzymes such as nitric oxide synthase and COX.^172^ Dienogest is a pregestational steroid of NF‐κB inhibitor, and it inhibits IL‐8 production to attenuate NF‐κB activation in endometriotic stromal cells in vitro.^50^

#### 3.1.3 MAPK/MEK/ERK

In EM, MAPK is activated to mediate the intracellular transmission of extracellular signals and induce cellular processes,^127^ as shown by a high phosphorylated extracellular signal‐regulated kinase (ERK) level.^173^, ^174^ RAS binds to RAF and activates ERK kinase (MEK1/2) to phosphorylate ERK, which is a major MARK signaling cascade.^127^ ERK1/2 regulates c‐fos and c‐jun expression to regulate mitosis and cell viability in endometrial cells under EM.^174^ E_2_, IL‐1β, and TNF‐α stimulated the phosphorylation of ERK1/2 in endometriotic stromal cells, but not in normal endometrial cells.^175^ Protease‐activated receptor 2 also activated ERK1/2 in cultured ectopic endometrial stromal cells.^176^

Sorafenib has completed phase IV clinical trials in several types of carcinoma, and it significantly abrogated the phosphorylation of RAF kinase by 64% via the MAPK/ERK pathway in stromal cells of EM patients. However, weight loss was observed in the xenograft EM mouse models.^87^ Vemurafenib is FDA‐approved for the treatment of metastatic melanoma and significantly inhibits ERK phosphorylation by over 60% in both endometriotic stromal cells and epithelial cells.^89^ U0126 is an MEK1/2 inhibitor that increases progesterone receptor (PR)‐αβ levels in endometriotic stromal cells.^91^ However, although the above treatments were shown to have significant efficacy, treatment of EM with MAPK inhibitors induced adverse effects on reproductive functions in animal models, including ovulation inhibition, embryotoxicity, and teratogenicity.^127^ Puerarin is a natural product that strongly binds to ERs; its binding affinity to ERs is one‐third that of E_2_, and it suppresses E_2_‐induced endometriotic stromal cells by 30% via the ERK pathway in vitro,^93^ and results in reduced adverse effects.

#### 3.1.4 PI3K/Akt/mTOR

PI3K phosphorylates phosphatidylinositol 4,5‐bisphosphate (PiP2) into phosphatidylinositol 3,4,5‐trisphosphate (PiP3) and activates protein kinase B (Akt).^177^ Mammalian target of rapamycin (mTOR), a downstream protein kinase of Akt, is overexpressed in ectopic lesions.^178^, ^179^ Reduction of the phosphatase and tensin homolog deleted from chromosome 10 (PTEN) by mutation^180^ enhanced the phosphorylation of Akt, thus promoting proliferation, inhibiting apoptosis, and reducing PR expression in EM.^91^ MK2206, an Akt inhibitor, is a drug candidate for cancer treatment that acts by increasing PRβ and PRαβ levels and decreasing the viability of endometriotic stromal cells, without affecting normal cells.^91^ WIN 55212–2 is a nonselective cannabinoid agonist that binds to cannabinoid receptor (CB)1 or CB2 to inhibit Akt levels and Akt phosphorylation, suggesting the inactivation of the Akt pathway. However, although it reduced the proliferation rate of endometriotic cells, it also reduced that of eutopic endometrial stromal cells.^98^

#### 3.1.5 Hippo/Yes‐associated protein (YAP)

The Hippo/YAP pathway is important for balancing cell proliferation and apoptosis. Upregulation of this pathway increased the viability of endometriotic cells, whereas knockdown of YAP increased apoptosis and decreased B‐cell/B‐cell lymphoma 2 (Bcl‐2) expressions.^181^ Verteporfin, a YAP1 inhibitor, inhibited the proliferation of endometriotic stromal cells, production of E_2_, and infiltration of immune cells.^75^ It is an FDA‐approved drug for the treatment of subfoveal choroidal neovascularization. EM mouse models showed decreased vessel tube formation and cell migration, with no reported effects on reproductive organs, infertility, or transgenerational influence.^75^ YAP1 is a potential target protein but has not been widely studied in EM.

#### 3.1.6 Metabolic process

The metabolic pathways toward increased lactate and dysregulation of glycolysis were shown as contributing factors for cancer progression.^182^ Lactate induces angiogenesis and supplies nutrients to proliferate tumor cells.^182^, ^183^ In EM peritoneal mesothelial cells, increased glycolysis, decreased mitochondrial respiration, decreased pyruvate dehydrogenase activity, and increased lactate was also observed.^46^ Dichloroacetate, a nonhormonal treatment or recurrence prevention of EM, reversed the pathophysiology of EM by inhibiting pyruvate dehydrogenase kinase to activate pyruvate dehydrogenase.^46^ Although dichloroacetate has completed a phase III clinical trial for lactic acidosis in 1998 and has widely studied in cancer, it has not been approved by the FDA for therapeutic use in cancer.

### 3.2 Proapoptotic mechanism

Apoptosis is a programmed cell death process that maintains the balance between the growth and differentiation of cells for tissue renewal. It is regulated by selective chromatin internucleosomal cleavage to shrink the cells.^184^ Apoptosis is important in the normal endometrium to remove dysfunctional cells and repair tissues during the menstrual cycle.^185^ Apoptotic cells were found to be more predominant in the endometrial epithelium glands than in the stroma.^186^ Cell apoptotic activity was found to be relatively low in EM.^187^ This can be explained by the reduced expression of proapoptotic factors (e.g., Bcl‐2‐associated X [Bax] and Bcl‐2 associated agonist of cell death [Bad]), overexpression of antiapoptotic factors (e.g., Bcl‐2), and dysregulation of cell cycle.^188^, ^189^ Endometriotic cells could not express surface receptors to trigger proapoptotic proteins; neither apoptotic signals were appropriately transduced, leading to proliferation being triggered instead.^188^ GnRH agonists, such as leuprolide acetate or the preclinical nonhormonal drug propofol, increased the levels of proapoptotic proteins, as well as decreased the levels of antiapoptotic proteins and promitogenic cytokines.^52^, ^100^ Melatonin is highly effective in amplification of apoptotic activity via regulating MMP‐3 signal, was able to regress EM at either early or late stage. Melatonin in high dose and long‐term treatment shows no adverse effects in EM rodents.^63^

#### 3.2.1 MAPK, Akt, and NF‐κB pathways

The signaling pathways MAPK/ERK, PI3K/Akt, and NF‐κB also regulated apoptosis in endometriotic cells. ERK1/2 was activated as an antiapoptotic protein in eutopic and ectopic endometrial glands throughout the menstrual cycle.^174^ Non‐E_2_ targeted treatments, such as selective PGE_2_ inhibitors, inhibited PGE_2_ receptor (EP) 2 and EP4 to induce apoptosis via multiple pathways including ERK1/2, AKT, and NF‐κB. They enhanced apoptosis by approximately 50% in both epithelial and stromal cells; thus, owing to their efficiency as selective or combination inhibitors, they are ideally used to treat stage I and II EM.^95^ PGE_2_ inhibitors are preferred to COX‐2 inhibitors due to their fewer adverse effects and lack of hypoestrogenic effects.^95^

BAY11‐7085 is an NF‐κB inhibitor that inhibits cell viability and enhances apoptosis in endometriotic cells by seven fold but induced less profound results in normal endometrial cells.^62^ The activity of Bay11‐7085 has been shown in in vitro studies, but there has been no clinical study of its therapeutic potential. Ginsenoside Rg3, genistein, and curcumin are found in natural products, and they enhanced apoptosis via regulation of the NF‐κB pathway.^79^, ^85^ In addition, ginsenoside Rg3 neutralized the effect of TNF‐α to regulate proliferation.^79^ Genistein had a similar capability as a GnRH agonist, that is, inhibiting transforming growth factor (TGF)‐β to regulate NF‐κB. Expression of Bcl‐2 was suppressed, whereas that of Bax was enriched. It also reduced COX‐2 and PGE_2_ expression to levels comparable to those in the control group.^85^ Curcumin inhibited MMP‐3 and increased the Bax/Bcl‐2 ratio by upregulating tumor protein p53 (p53).^81^ These natural products are highly attractive owing to their potential efficacies and minimal side effects reported in in vitro and in vivo studies of EM and clinical studies of other diseases.

Akt is a pleiotropic regulator of apoptosis that increases endometriotic cell survival and decreases apoptosis.^190^ Akt/mTOR can be inhibited by endoplasmic reticulum stress.^51^ Tunicamycin enhanced TNF‐related apoptosis‐inducing ligand‐induced apoptosis by inducing endoplasmic reticulum stress in endometriotic stromal cells. The effect was more potent in endometriotic cells than in eutopic endometrial cells^73^; however, its action mechanism and pharmacokinetics profile have yet to be elucidated.

#### 3.2.2 E_2_/TNF‐α

Transcription of antiapoptotic Bcl‐2 protein was increased by E_2_ through the promotion of thymic stromal lymphopoietin.^191^ Extranuclear kinases are activated by an elevated ER complex to trigger a rapid nongenomic signaling cascade and inhibit apoptosis in stromal and epithelial cells.^192^, ^193^ Genistein interfered with the E_2_/ER pathway to induce apoptosis and apoptotic proteins in endometrial hyperplasia.^194^ Moreover, the activity of E_2_ strongly regulates TNF‐α‐induced effects. In a healthy endometrium, TNF‐α stimulated apoptosis. In eutopic and ectopic endometriotic cells, TNF‐α stimulated proliferation and inhibited apoptosis instead.^195^ In endometriotic lesions, apoptosis signal‐regulating kinase (ASK‐1), a TNF‐α induced apoptosis complex I, interacted with ERβ as well as serine/threonine kinase receptor‐associated protein (STRAP) and 14‐3‐3 proteins.^62^ Formation of this complex disrupted the association of TNF‐receptor‐associated factor 2 (TRAF2) and ASK‐1 for TNF‐α‐induced apoptosis.^196^, ^197^ TNF‐α‐induced apoptosis complex I, complex II, and apoptosome were all inhibited, which dysregulated apoptosis and activated the invasiveness of lesions for survival. In addition, in endometriotic tissue, TNF‐α from macrophages and natural killer (NK) cells induced the generation of the steroid receptor coactivator (SRC)‐1 isoform from cleaved MMP‐9.^198^ ERβ interacted with the caspase (CASP)‐8 and SRC‐1 isoforms to prevent activation of TNF‐α‐induced apoptosis complex II in endometriotic lesions in ectopic sites.^62^ Therefore, inhibition of ASK1/ERβ/STRAP‐14‐3‐3 and ERβ/CASP8/SRC‐1 protein complex are potential therapeutic targets to regulate apoptosis via the E_2_/ER/TNF‐α pathway.

### 3.3 Autophagy mechanism

Autophagy is a process related to nonapoptotic cell death and is defined as self‐degradation. It balances the energy sources by removing misfolded proteins, damaging organelles, and eliminating intracellular pathogens. It promotes the proteolytic degradation of cytosolic components at the lysosome.^199^ Recently, there have been more studies on the role of autophagy in both accelerating and decelerating the pathogenesis of EM. To examine the pathophysiology of autophagy in regulating EM, we divided the section into antiautophagy and proautophagy to discuss the controversies in the progression of EM.

#### 3.3.1 Antiautophagy, anti‐EM

Downregulation of apoptosis favors the stimulation of autophagy, thus promotes EM growth.^200^ A significant reduction in apoptosis inducer p53 mediated by Akt, an increased lapidated microtubule‐associated protein 1A/1B light chain 3 (LC3)‐II, and a significant decrease in sequestosome 1 (SQSTM1) were observed in ovarian endometrioma.^200^ LC3‐II is a standard autophagy marker while SQSTM1 is an autophagy adaptor protein that transfers ubiquitinated proteins to the autophagic machinery and is degraded via autophagy to indicate the activation of autophagic flux.^197^, ^198^, ^199^, ^200^, ^201^ Hypoxia upregulated autophagy in endometriotic cells to induce HIF‐1α.^202^ Overexpression of HIF‐1α under normoxic conditions also induced autophagy.^203^ Decreased expression of homeobox A10 (HOXA10) induced autophagy in EM, which was attributed to excessive inflammation.^204^ It contributed to mitochondrial damage, increased PGE_2_, and increased mitochondrial ROS,^203^, ^204^ all stimulated autophagic processes. An increased oxidant heme oxygenase (HO)‐1 was observed in ovarian endometrioma to activate an adaptive defense mechanism and negatively modulate inflammation and apoptosis, and positively stimulated autophagy.^200^ A combination therapy with MK2206 and chloroquine was found to be more effective than with either MK2206 or chloroquine in reducing endometriotic cell viability and preventing regrowth by inhibiting autophagy.^102^ SQSTM1 expression was significantly upregulated.^102^

#### 3.3.2 Proautophagy, anti‐EM

Upregulation of autophagy promoted apoptosis and suppressed cell growth and invasion in EM.^205^, ^206^ Endometriotic stromal cells have an abnormal response to progesterone, which suppresses PTEN expression, suppresses autophagy, and reduces apoptosis in the menstrual cycle via the AKT/mTOR pathway.^207^ Increased expression of YAP significantly decreased autophagy through the mTOR pathway in eutopic endometrium stromal cells.^208^ Mullerian‐inhibiting substance (MIS) induced autophagy and apoptotic cell death and inhibited proliferation in vitro.^104^ MIS, also known as anti‐Müllerian hormone, however, was found to be increased in EM lesions and in the serum of women with ovarian endometrioma and promoted inflammation. More preclinical data are required before the clinical application of this agent in EM treatment.^209^, ^210^ A ginsenoside metabolite, protopanaxadiol (PPD), reduced ERα expression and induced PRα expression in vitro and in vivo, which then induced autophagy and suppressed lesion growth, resulting in a significantly different expression of autophagy‐related genes, including downregulated estrogen receptor (ESR1), SQSTM1, and TGF‐β levels as well as upregulated CASP‐3, ATG ‐3/‐5/‐12 after treatment.^103^

### 3.4 Anti‐Cell migration and invasion mechanism

Cell migration and invasion are critical processes for EM establishment according to the implantation theory. EM is believed to occur due to the shedding of endometrial cells and then migration to ectopic sites.^15^, ^211^ Migration of endothelial cells mediates angiogenesis and plays a role in the pathophysiology of EM.^212^

#### 3.4.1 Wnt/β‐catenin

The wingless‐type mouse mammary tumor virus integration site family (Wnt) plays a role in developmental processes and homeostasis. β‐catenin is crucial in regulating the cell cycle, which includes proliferation, differentiation, and migration in ectopic lesions.^213^ In the presence of Wnt ligands, an accumulation of β‐catenin translocates to the nucleus and interacts with T‐cell factor/lymphoid enhancer‐binding factor (Tcf/LEF) transcription factors to activate the Wnt/β‐catenin signaling pathway.^107^ Wnt/β‐catenin was found to be abnormally activated in EM.^214^ Multi‐drug resistance protein 4 (MRP4) regulates Wnt/β‐catenin signaling by stabilizing β‐catenin activity. It was involved in the pathogenic transformation of EM endometrium, confirmed in the ectopic lesion. In MRP4‐knockdown endometrial epithelial cells, reduced activity of β‐catenin was found to downregulate Wnt/β‐catenin signaling.^215^

Overexpression of T‐cadherin inhibited the invasion and migration of cells in EM, and the phosphorylation of heat shock protein (HSP)‐ 27 and c‐Jun N‐terminal kinase (JNK)‐1/2/3 was promoted. MMP‐2/‐9 and vimentin expression was lowered in endometriotic cells.^216^ MMP‐2/‐9 and Cyclin D1 are targets of Tcf/β‐catenin genes and were found to be upregulated in endometrial epithelial or stromal cells of EM,^107^ MMPs are responsible for regulating migration, invasion, and angiogenesis by balancing growth factors and cytokines, and high expression of MMPs in EM favors lesions.^217^ PKF115‐584 and CGP049090 are fungal derivatives and were screened through high‐throughput assay to disrupt Tcf/β‐catenin complex,^218^ significantly inhibiting MMP‐9 activity and cell invasiveness in epithelial and stromal cells to a level close to that of normal endometrium.^108^ However, they have not been widely studied in EM. Genistein regulates invasion and migration through downregulation of MMP‐2/‐9 by targeting NF‐κB, as shown in an in silico study and in an EM mouse model.^112^ In addition, Wnt/β‐catenin is important for stem cell maintenance and tissue homeostasis^107^; thus, an antagonist of Wnt/β‐catenin may have potential side effects. Moreover, Wnt2 is secreted by ectopic stromal cells, which induces β‐catenin signaling activity in ectopic endometrial epithelial cells, as well as expressions of the growth‐associated proteins in endometrial epithelial cells.^48^ Wnt2/β‐catenin is a pathway involved in the communication of stromal and epithelial cells in EM, which can be modified by metformin.^48^

#### 3.4.2 Rho/ROCK

Ras homolog family member A/Rho‐associated coiled‐coil kinase (RhoA/ROCK) plays a major role in cell migration via phosphorylation of cytoskeletal regulatory proteins and results in actin depolymerization, actomyosin contraction, endothelial cell adhesion, and migration.^219^ ROCK in the endothelial cytoskeleton is activated by proangiogenic stimuli.^219^ VEGF stimulates RhoA/ROCK and mediates endothelial cell migration.^220^ ROCKII regulates cell body contraction during migration.^221^ It is a positive regulator of p27 to further activate cell migration in endometrial stromal cells and regulate RhoA in the cells.^222^ Fasudil is a ROCK inhibitor used clinically and has the potential for treating EM by reducing endometriotic cell viability and inducing apoptosis by targeting Rho/ROCK.^115^

### 3.5 Anti‐Fibrotic mechanism

Recently, EM was defined as a profibrotic condition, as a new concept by Vigano et. al.^223^, ^224^ The crucial role of fibrosis and differentiation of myofibroblasts in the progression of EM lesions have been reported. Fibrosis is the pathological activity when activated myofibroblasts accumulate leading to contraction of the collagenous extracellular matrix and anatomical structure disruption. The event of fibrosis justifies EM‐associated morbidity and adhesiveness and is considered a potential EM therapeutic target.^223^, ^224^

#### 3.5.1 TGF‐β mediated pathways

TGF‐β1 is a stimulating factor that triggers the production of collagen and transition of epithelial to mesenchymal phenotype, leading to fibrosis. TGF‐β1 was found to be significantly increased in the peritoneal fluid of women with EM.^225^ Wnt signaling is required for TGF‐β1‐mediated fibrosis.^226^ Matsuzaki and Darcha^110^ also showed that the Wnt/β‐catenin pathway was involved in fibrogenesis in EM. PKF1150‐584, CGP049090, and EGCG significantly inhibited TGF‐β1‐induced fibrotic markers, including α‐smooth muscle actin (α‐SMA), type I collagen, fibronectin, and connective tissue growth factor in stromal cells via Wnt/β‐catenin signaling, with EGCG showing the greatest effects.^110^, ^111^ ICG‐001 and C‐82 are CBP inhibitors. They are also metabolites of PRI‐724, which is under clinical trials for its antitumor activity,^227^ they inhibited fibrosis via Wnt/β‐Catenin signaling, with ICG‐001 showing great efficacy in upregulating apoptosis and inhibiting migration as well as C‐82 showing potent inhibition of proliferation and cell viability.^106^

#### 3.5.2 Rho/ROCK

Activation of the Rho/ROCK signaling pathway was associated with fibrosis in EM.^228^ Heparin has completed phase IV clinical trials for several medical indications, including anticoagulation and cancer. It inhibited RhoA, ROCKI, ROCKII, and α‐SMA expression and activated the Rho/ROCK pathway to attenuate endometriotic stromal cell contractility, differentiation, and fibrosis.^117^

### 3.6 Antiangiogenesis mechanism

Angiogenesis is highly regulated in the female reproductive system, and it is a process that results in the formation of new blood vessels from existing ones. Three mechanisms of angiogenesis have been described as sprouting, elongation, and intussusception.^229^ It provides neovascularization to deliver essential nutrients and oxygen supply for the growth of endometriotic lesions.^230^

#### 3.6.1 VEGF

VEGF regulates angiogenic, endothelial cell‐specific mitogenic, and vascular permeability activities of endometrial and endometriotic cells through vascular endothelial growth factor receptor (VEGFR) 1–3 on the microvascular endothelial cell surface. The VEGF family helps in establishing and maintaining endometriotic foci.^231^, ^232^, ^233^ VEGFR2 is a highly active kinase that plays a major role in angiogenesis. VEGF binds to two proximal VEGFR2 receptors, promotes vascular permeability, increases the migration and proliferation of endothelial cells, and contributes to the formation of new blood vessels.^234^, ^235^ Sunitinib, SU6668, SU5416, sorafenib, and pazopanib were originally indicated as anticancer drugs but were further repurposed and tested for efficacy against EM.^119^, ^120^, ^123^, ^126^, ^128^ SU5416 selectively bound to VEGFR, and only reduced graft size by 5%.^123^, ^126^, ^236^ SU6668, a multi‐kinase inhibitor, reduced the endometrial graft by 25% by blocking VEFFR‐2, fibroblast growth factor receptors (FGFR)‐1, and platelet‐derived growth factor receptors (PDGFR)‐β.^123^, ^236^ Sunitinib regulates angiogenesis and apoptosis through multi‐kinase inhibition, regressing 50% cyst in an EM rat model.^120^ The effects of pazopanib, sunitinib, and sorafenib on VEGF/VEGFR protein kinase pathways and their actions in EM were compared by Yildiz et. al.,^128^ which showed that pazopanib had better efficacy than the control and other treatments, reducing EM lesions by at least 45%, but Sorafenib was better in regulating VEGF.^128^ However, tyrosine kinase inhibitors that regulate VEGFR are associated with a significant risk of treatment toxicities.^237^ In sarcoma, pazopanib treatment led to a higher incidence of adverse effects, including fatigue and hypertension, compared with sunitinib or sorafenib.^237^ An alternative VEGF regulator, EGCG, significantly inhibited lesion growth by suppressing VEGFC/VEGFR2 signaling. Overexpression of VEGFC induces migration of endothelial cells, increases vascular permeability, and induces angiogenesis and endometriotic lesions growth.^70^, ^129^ Prodrug of EGCG (ProEGCG), reduced lesion size, weight, and VEGF concentrations in plasma to a greater extent than the parent EGCG molecule. More importantly, there were no signs of side effects on reproductive tissues.^130^ Quinagolide, a dopamine receptor 2 agonist, is under phase II clinical trial to examine its efficacy in reducing EM lesion size and related pain. It can completely reverse the size of lesions by downregulating the VEGF/VEGFR2 pathway in EM.^135^ It has an acceptable safety profile and does not stimulate serotonin receptor subtype 2b to proliferate fibroblasts in cardiac valve tissues, and thus holds great potential as an alternative of tyrosine kinase inhibitor for EM.^135^

Under normoxic conditions, HIF‐1α is regulated via proteasome‐mediated degradation.^238^ However, under hypoxic conditions, HIF‐1α escapes ubiquitination and binds to hypoxia‐responsive enhancer on VEGF genes to upregulate their expression.^239^ HIF‐1α was upregulated in lesions, thus promoting VEGF expression in an EM mouse model.^240^ Increased VEGF secretion was observed in hypoxia‐induced endometrial stromal and glandular cells compared with that under normoxic conditions.^241^ Oxidative stress also increased VEGF secretion, as shown by the results obtained after incubating endometrial epithelial cells with oxidized low‐density lipoprotein.^242^ In the EM peritoneal environment, PGE_2_ was upregulated to elicit cell signals through upregulation of VEGF and FGFR. It induced the expression of COX‐2 and synthesis of E_2_ in ectopic endometrial cells to increase the production of MMP, thus enhancing VEGF expression and inducing angiogenesis.^229^, ^243^ TNF‐α mediates the angiogenic activity of macrophages, which stimulates endothelial cell migration and induces the release of VEGF and the formation of bloodvessel.^244^ Pyrrolidine dithiocarbamate inhibited NF‐κB activation and attenuated TNF‐α‐mediated VEGF and MMP‐9 expressions.^132^ Pentoxifylline attenuated TNF‐α mediated effects in other diseases, requires further investigation of this in EM, but it suppressed angiogenesis by reducing VEGFC and VEGFR2 (Flk‐1) expression levels in glandular cells of endometriotic lesions.^133^ Pentoxifylline is an immunomodulatory agent and has completed a phase III clinical trial of EM‐associated infertility. The clinical trial did not present any data on lesion progression or recurrence, only on pregnancy rate.^245^

#### 3.6.2 Rho/ROCK

The Rho/ROCK pathway regulates VEGF‐mediated endothelial cell activation and vessel stability.^233^, ^246^ RhoB mitigates VEGF‐induced vessel sprouting via the RhoA/ROCK signaling pathway.^247^ RhoA/ROCK activity can be blocked by inhibiting protein prenylation in endothelial cells to reduce migration and adhesion.^248^ Avian myelocytomatosis virus oncogene cellular homolog (C‐Myc) is a target of the PI3K/Rho/ROCK signaling pathway and regulates VEGF expression. Under hypoxic conditions in EM, guanosine triphosphate (GTP)‐bound Rho was regulated in a PI3K‐dependent manner to induce VEGF by binding to C‐Myc without suppressing the induction of HIF‐1α.^249^ ROCK activation initiates E_2_‐induced angiogenesis.^219^ Inhibitors that target the Rho/ROCK pathway should be further investigated for their ability to regulate angiogenesis, migration, invasion, and fibrosis of endometriotic cells.

### 3.7 Antioxidative stress mechanism

Oxidative stress is the imbalance between ROS production and antioxidant function, which plays a main role in EM progression.^250^ ROS are molecules that have unpaired electrons and can damage lipids, nucleic acids, and proteins.^251^, ^252^ Apoptotic endometrial tissues and macrophages induced oxidative stress in EM through retrograde menstruation.^253^ Oxidative stress causes DNA hypermethylation and histone modification, which are linked to aberrant endometrium development in EM.^254^

#### 3.7.1 ROS‐mediated pathway

The production of ROS in the progression EM could be achieved through several pathways, including activation of inflammatory cytokines, MMPs, and transcriptional factors, such as NF‐κB.^255^ Environmental factors, such as reproductive toxins, can also increase oxidative stress and decrease the expression of antioxidant enzymes.^256^ Di‐2‐ethylexyl phthalate is used as a plasticizer and solvent in cosmetic and consumer products, and they altered the NF‐κB signaling pathway and expression levels of ER and PR in human endometrial stromal cells via activation of the MAPK/ERK and PI3K/Akt signaling pathways.^257^

Balancing ROS production and antioxidant function by inhibiting free radicals or increasing antioxidant levels is important to regulate oxidative stress. Antioxidants include the enzymes superoxide dismutase (SOD), glutathione peroxidase (GPx), HO, and catalase, as well as nonenzymatic molecules such as vitamins A, C, and E.^250^, ^252^, ^255^ Significantly lower levels of antioxidants were found in the peritoneal fluid of EM, which indicated that women with EM had a low free radical‐scavenging ability.^255^, ^258^, ^259^ N‐Acetyl cysteine (NAC) is an antioxidant that inhibits ROS as well as abrogates ERK activation and proliferation.^137^ EGCG and resveratrol are both well‐known natural antioxidant supplements for the treatment of EM and other diseases, including cancer.^130^, ^138^, ^260^ Resveratrol acts as a radical scavenger, and it significantly regressed endometriotic implants, decreased lipid peroxidation, and increased at least 50% endogenous antioxidant capacity in tissues and serum in EM.^138^ The antioxidant effects of ProEGCG against EM were significantly greater than those of EGCG and at least four fold that of the control.^130^ Melatonin reduced the level of oxidative stress markers and increased level of antioxidants in EM. EM implants in rat were significantly regressed.^142^, ^143^ Caffeic acid found in plants exerted similar antioxidant effects in EM and an enhanced nuclear translocation of nuclear factor erythroid 2–related factor 2 (Nrf2) regulated antioxidant enzymes in EM.^141^, ^261^

#### 3.7.2 NO‐mediated pathway

NO is a vasodilator that mediates endothelium‐dependent vasodilation and angiogenesis.^262^ NO has an unpaired electron and is a highly reactive free radical. The formation of NO requires NO synthase (NOS), l‐arginine, oxygen, and a number of cofactors, including nicotinamide adenine dinucleotide phosphate, flavin mononucleotide (FMN), and flavin adenine dinucleotide.^263^ Macrophages increased IL‐10 in EM and stimulated NO^264^; NO and NOS levels were increased in endometrial tissues in EM.^265^ Increased E_2_ level activated the formation of NO,^266^ implying that macrophages and E_2_ regulate NO‐mediated oxidative stress in EM.

#### 3.7.3 Iron‐mediated pathway

Iron carries hemoglobin throughout the body, an overproduction of it not only enhances epithelial cell proliferation but also induces oxidative stress.^267^, ^268^ Ferritin is a cellular iron storage that generates hydroxyl radical via Fenton reaction to initiate a free radical chain reaction, namely lipid peroxidation.^37^, ^269^ McKinnon et al.^37^ reviewed studies on the implication of mTOR on iron homeostasis and suggested the dysregulation of mTOR in EM could overload iron levels to stimulate oxidative stress. Currently, there is lack of pharmaceuticals targeting iron‐mediated oxidative stress in EM and regulating the mTOR signaling pathway might reduce oxidative stress.

### 3.8 Immune system and inflammation

Immune system dysregulation and chronic inflammatory response are characterized in EM.^270^, ^271^ The adaptive immune system with increased quantity of regulatory T (Treg) cells and a shift towards type 2 immune response fail to recognize the endometriotic cells in the peritoneal cavity.^272^ On the contrary, the innate immune system in EM is characterized by an enhanced activation state of macrophages, along with upregulated cytokines, but downregulated phagocytosis,^273^ as well as a reduced cytotoxicity of natural killer (NK) cells,^274^ and an altered population of dendritic cells.^275^ These promote inflammation and contribute to the implantation process in EM and new drugs that modulate these specialized cells hold promise as a novel immunotherapy for EM.

#### 3.8.1 Macrophages and cytokines

Macrophages exert their inflammatory effects against tumors via host defense mechanisms.^276^ IL‐1 is a proinflammatory cytokine secreted by activated monocytes, macrophages, or NK cells, and is responsible for activating lymphocytes to reduce immune surveillance and stimulate PGE_2_ via COX‐2 in EM stromal cells.^271^, ^277^, ^278^ IL‐6 is responsible for stimulating B‐cell activity and T‐cell differentiation. The levels of IL6 in serum and peritoneal fluid are high, but its receptor is reduced in EM. However, endometriotic cells are resistant to its growth‐inhibitory effects.^271^, ^279^, ^280^ VEGF and TNF‐α are proinflammatory cytokines secreted by activated lymphocytes, neutrophils, NK cells, and macrophages to initiate the inflammatory cascade.^271^

Niclosamide is an FDA‐approved nonsteroidal therapy for antihelminth^281^ and was found to inhibit the proliferation and growth of endometriotic lesions. It reduced MAPK, WNT, and inflammation signaling‐related genes, such as NF‐κB and signal transducer and activator of transcription 3, in an EM mouse model. No disruption to reproductive function was observed, indicating potential therapeutic efficacy and safety for EM treatment.^154^ NAC regressed lesions by suppressing COX‐2 and MMP‐9 expression. Its side effect is mild and seemed to not interfere with fertility in vivo.^144^ Crocin, curcumin, and metformin inhibit proinflammatory cytokines and chemokines, including TNF‐α, IL‐1β, IL‐6, VEGF, and so forth.^148^, ^149^, ^155^ These are responsible for recruiting and activating macrophages, neutrophils, and NK cells to the EM site and further enhancing angiogenesis and inflammation.^155^, ^282^ Acai, a natural product found in plants from the Amazon region, has completed phase III clinical trials as an antioxidant agent. It reduces EM lesions by targeting active macrophages, VEGF, and COX‐2.^156^ Resveratrol inhibited inflammatory responses by reducing peritoneal and serum cytokines,^150^ as well as activating sirtuin 1 (SIRT1) to significantly suppress IL‐8 in TNF‐α‐induced endometriotic stromal cells via NF‐κB.^151^ SIRT1 has a dual function as a tumor suppressor or promoter,^283^ and it is a potential target protein, considering that it is a strong regulator of the inflammatory responses, apoptosis, and oxidative stress in EM.^284^ Macrophage migration inhibitory factor (MIF) is a proinflammatory cytokine that is upregulated in peritoneal fluid in women with EM. It activates the MAPK/ERK pathway, stimulates COX‐2, and produces PGE_2_ in ectopic endometrial cells. MIF also contributes to angiogenesis via its effect on endothelial cell proliferation.^285^ ISO‐1 is a MIF antagonist and a leading molecule discovered to treat sepsis.^286^ It inhibited angiogenic and proinflammatory pathways via VEGF/VEGFR in peritoneal EM in vivo, without interrupting the reproductive cycle.^287^

#### 3.8.2 Estrogen

In EM, increased ESR2/E_2_ induces COX‐2 and PGE_2_, upregulates macrophages and NF‐κB,^288^, ^289^ and leads to oxidative stress.^255^ ERβ modulates macrophage infiltration via NF‐κB in EM^290^ and induces IL‐1β by interacting with the inflammasome complex to evade immune surveillance and promote the attachment of lesions at the endometriotic sites.^196^ E_2_ activates thymic stromal lymphopoietin and induces the secretion of endometrial stromal cells‐associated growth‐promoting cytokines, including monocyte chemoattractant protein 1 and IL‐8, via the JNK and NF‐κB pathways.^291^ IL‐6 reduces E_2_ production in human granulosa tumor cells via the MAPK signaling pathway,^292^ implying its possible targeting of E_2_ biosynthesis in EM. Puerarin reduced the level of ERβ, but not ERα, by inhibiting P450AROM. In a rat model, simultaneous reduction of E_2,_ COX‐2, and PGE_2_ expression levels, as well as enhancement of the metabolism of E_2_ into estrone,^152^ led to the inhibition of lesion growth in the ectopic endometrium tissues. In another study, ginsenoside PPD inhibited the E_2_ signal, thus activating the cytotoxicity of NK cells against ectopic endometrial stromal cells to regulate cell death. This was also confirmed in peritoneal fluids of the EM mouse model.^103^

## 4 POTENTIAL PHARMACEUTICALS WITH MULTIPLE TARGETS

In the treatment of EM, targeting a specific pathway, or multiple pathways alleviate the lesions. Targeting a single molecule can lead to several anti‐EM effects, as downstream transduction elements are usually connected to a series of molecular events as secondary responses. However, owing to synergistic effects, a multiple target therapy may have a greater suppressive effect on lesions compared with a single targeted therapy.^196^ Table 4 summarizes several single pharmaceuticals with multiple molecular targets, which affect multiple signaling pathways in a complex disease such as EM.

**Table 4:** Pharmaceuticals that hold multiple molecular targets to different pathophysiology for endometriosis treatment

Pharmaceuticals	Classification	Proliferation	Apoptosis	Autophagy	Cell migration	Cell Invasion	Fibrosis	Angiogenesis	Oxidative stress	Immune and inflammation
Melatonin	Anxiolytic agent	‐	MMP‐3^63^	‐	‐	‐	‐	VEGF, MMP‐9^142^	ROS^143^	COX‐2^143^
Metformin	Antidiabetic agent	‐	‐	‐	‐	Wnt2^48^	‐	‐	‐	Cytokines^149^
NAC	Amino acid cysteine	‐	‐	‐	‐	‐	‐	‐	ROS^137^	Cytokines^144^
Curcumin	Curcuminoid	‐	MMP‐3^81^	‐	‐	‐	‐	Chemokine and cytokines^253^	‐	Chemokine and cytokines^253^
EGCG	Catechin	E_2_ ^167^	‐	‐	TGF β1^110^	TGF β1^110^	TGF β1^110^	VEGFR2^70^, ^129^	ROS^130^	‐
		TGF β1^110^								
		VEGFR2^70^, ^129^								
Genistein	Isoflavone	‐	TGF‐β^85^	‐	NF‐κB^196^	NF‐κB^196^	‐	‐	‐	‐
Ginsenoside	Steroid glycoside	TNF‐*α* ^79^	TNF‐*α* ^79^	ERα and PRα^103^	‐	‐	‐	TNF‐α ^79^	‐	Induce NK cells toxicity^103^
Puerarin	Isoflavone	ERs^93^	‐	‐	‐	‐	‐	‐	‐	P450AROM^152^
Resveratrol	Phytoalexin	ESR1^66^	‐	‐	‐	‐	‐	Cytokines^150^	ROS^138^	Cytokines,^150^ SIRT1^151^

Abbreviations: AKT, protein kinase B; AMPK, adenosine monophosphate‐activated protein kinase; CASP, caspases; CHOP, CCAAT/enhancer‐binding protein homologous 10 protein; COX, cyclooxygenase; DPPH, 2,2‐diphenyl‐1‐picrylhydrazyl; E_2_, Estrogen; EGCG, epigallocatechin gallate; ER, estrogen receptor; ERK, extracellular signal‐regulated kinase; ESR1, estrogen receptor 1; HMGB1, high mobility group box 1; H_2_O_2_, hydrogen peroxide; IKKB, IκB kinase beta; MAPK, mitogen‐activated protein kinase; MMP, matrix metallopeptidases; NAC, N‐acetyl cysteine; NF‐κB, nuclear factor κB; NK cells, natural killer cells; NOD2, nucleotide‐binding oligomerization domain‐containing protein 2; Nrf2, nuclear factor erythroid 2–related factor 2; O_2_, oxygen; OH, hydroxide; P450AROM, aromatase; PI3K, phosphoinositide 3‐kinases; PR, progesterone receptor; REDD1, protein regulated in development and DNA damage response 1; ROS, reactive oxidative stress; SIRT1, sirtuin 1; TCM, traditional Chinese medicine; TGF, transforming growth factors; TNF, tumor necrosis factor; VEGF, vascular endothelial growth factor; VEGFR, vascular endothelial growth factor receptor; Wnt, wingless‐type mouse mammary tumor virus integration site family.

### 4.1 Hormonal pharmaceuticals

Melatonin is a natural substance produced by plants. It is also a hormone produced in the pineal gland to regulate neuroendocrine functions and inhibits LH and FSH secretion from the anterior pituitary gland.^293^ Melatonin acts as an antioxidant and anti‐inflammatory agent and is currently under phase 2 clinical trial for reducing EM‐related pain. Another randomized, double‐blind, and placebo‐controlled clinical trial of melatonin was completed in 2013. The results of the study showed that melatonin acts as an analgesic and can relieve EM‐related chronic pain.^294^ Melatonin receptor (MR)1A and MR1B are significantly upregulated in peritoneal EM lesions compared with those in the eutopic tissue. Melatonin has been shown to reduce EM lesions in various studies. It inhibits cell proliferation and modulates endometrial epithelial cell function.^295^ Melatonin also inhibits angiogenesis via VEGF and oxidative stress via regulating radical scavenging activity and amplifies apoptotic activity via CASP3 mediated pathway in vivo and *in vitro* in EM.^63^, ^142^, ^143^ Melatonin has no adverse effects on reproductive functions, instead, it can improve ovarian functions, and thus has potential to treat EM‐related infertility.^296^, ^297^ High‐dose intravenous treatment of pain and sepsis with melatonin showed no adverse effects.^298^ Its bioavailability is 15%.^299^ Long‐term therapeutic investigation of melatonin in EM should be conducted to elucidate its ability to regulate E_2_ functions in EM.

### 4.2 Nonhormone pharmaceuticals

Metformin was shown to target multiple pathways^300^ by regulating stromal‐epithelial cell communication in EM via Wnt2‐mediated signaling^48^ and exerted an anti‐inflammatory effect through regulating cytokines.^149^ Although a mild side effect was implied,^149^ metformin regulated reproductive functions,^301^ and improved conception in EM patients by inhibiting serum cytokine production.^149^ Metformin is available in the market as a treatment for type 2 diabetes and PCOS in women. Considering its low cost, metformin was advocated to be used as a long‐term treatment.^302^ NAC, an acetylated form of cysteine, has been prescribed as an antidote since the 1960s. It replenishes intracellular glutathione levels and modulates the redox environment; therefore, NAC is a strong antioxidant.^303^ In EM, it acts as an antioxidant, antiproliferative, anti‐inflammatory, and anti‐invasiveness agent via ROS‐scavenging mechanism or through regulating cytokines in vitro and in vivo.^137^, ^144^ NAC is highly efficacious at low doses, and with no adverse effects in EM.^144^ Long‐term adverse effects are also limited, including no effect on fertility.^137^, ^144^ NAC is considered to have a good safety profile and has been evaluated in phase 4 clinical trials for treating gastrointestinal and metabolic diseases.^303^, ^304^ Its pharmacokinetics and toxicity profiles are available; its terminal half‐life is 6.25 h after oral administration and bioavailability is 9.1%.^305^ NAC is commercially available and cost‐effective as a dietary supplement in the market; however, studies on its efficiency in EM are limited, requiring more preclinical evidence.

### 4.3 Natural products

Natural products have a long history of use in the management of medical conditions. Research advances in analytical and synthetic chemistry have improved the identification and isolation of active compounds from natural products. EGCG is a polyphenol catechin from green tea and a well‐known antioxidant. It exerts efficacy against diseases including cancer, diabetes, and inflammation.^306^ In EM, it exerts ant antiangiogenetic effect via the VEGFC/VEGFR2 pathways,^70^, ^129^ antioxidant effects via ROS‐scavenging mechanism,^130^ antiproliferative effect via reduction of E_2_ production, and anti‐migration and anti‐invasion effects via TGF‐β1‐induced phosphorylation of ERK1/2 and MAPK pathways,^167^ thus inhibiting the development and growth of lesions. Promising evidence of its high potency and efficacy, and without major side effects in reproductive functions were reported.^130^, ^167^ EGCG is currently under phase 2 clinical trial for reducing lesion size and pain as well as an evaluation of its safety profile in EM. On the contrary, EGCG act as an adjuvant that brings synergistic effects, as well as reduces adverse effects in cancer treatment.^307^ This suggests a potential role of EGCG in combination therapy with current EM treatment. However, the low bioavailability of EGCG has limited its attractiveness in the market.^308^ ProEGCG, is a prodrug of EGCG, shows higher bioavailability and greater efficiency than EGCG to reduce lesions in vivo.^130^ More studies should be conducted to confirm the underlying mechanism of ProEGCG in the treatment of EM. Resveratrol is a polyphenol found in grapes. In EM, it reduces proliferation via an anti‐E_2_ mechanism targeting ESR1,^66^ inhibits inflammatory responses via radical scavenging,^138^ and inhibits angiogenesis by reducing the cytokines COX‐2 and VEGF^150^ and activating SIRT1.^151^ Resveratrol has completed a phase 4 clinical trial in EM and is safe and effective in relieving EM‐related pain, as well as in reducing serum CA125 and prolactin levels. Resveratrol is well‐known for its chemopreventive property. It also has a promising clinical profile in cancer treatment, nevertheless, the rapid metabolism rate of resveratrol has limited its efficacy in vivo.^309^

Curcumin, genistein, ginsenoside, and puerarin are not under any EM clinical studies but have been clinically evaluated in breast cancers, endometrial carcinoma, endothelial functions, and so forth. They have been studied for their action mechanism against EM in primary cells, cell lines, and animal models. Curcumin, which is found in ginger and turmeric, enhances apoptosis by increasing the Bax/Bcl2 ratio through targeting of MMP‐3 via NF‐κB^81^ and regulates angiogenesis and inflammation by targeting chemokines and cytokines^155^ in EM. It has multiple biological effects in different diseases, including cancer and inflammatory diseases. It establishes a good safety profile with no acute toxicity.^310^ Genistein is an isoflavone that acts as an E_2_ agonist or antagonist to manage postmenopausal symptoms. In EM animal models, genistein downregulated MMP‐2/‐9 and regulated cell invasion and migration by targeting NF‐κB.^311^ It also regulated NF‐κB by inhibiting TGF‐β.^85^ Long‐term treatment with genistein lower the incidence of endometrial hyperplasia and provided support for bone formation in postmenopausal women.^83^, ^312^ It acts as a chemopreventive and chemotherapeutic agent against cancers and has synergistic effects with other anticancer drugs.^313^ Toxicity of high dose is minimal, so it needs more study to test the safe range.^314^ Ginsenoside RG3, extracted from ginseng, restored TNF‐α‐induced effects by inhibiting NF‐κB, VEGF, and CASP‐3 in EM, which are responsible for cell proliferation, apoptosis, and angiogenesis.^79^ Ginsenoside PPD regulated ERα and PRα expression to suppress autophagy and lesion growth in EM.^103^ It also targeted E_2_‐induced NK cell cytotoxicity to regulate the immune system in EM.^103^ Ginsenoside also possess synergistic effect with anticancer drugs, as well as prevents toxicity and morbidity from chemotherapy.^315^ Puerarin is a phytoestrogen, binds to ERs via the ERK pathway to regulate proliferation in EM.^93^ It also regulates inflammation in ectopic endometrium by inhibiting P450AROM and COX‐2 and promoting ERβ expression to facilitate E_2_ metabolism in EM.^152^ Its therapeutic effects are studied extensively in diseases including cancer and cardiovascular disease.^316^

Most of these products have known toxicity or pharmacokinetic profiles and act via multiple targets, making them beneficial as anti‐EM agents. However, their poor aqueous solubility and low oral bioavailability in vivo is the major challenge to be potential EM treatment.^82^, ^92^, ^315^ There are several approaches available currently to progress the bioavailability of drugs, which include prodrug approach,^130^ solid dispersions approach,^317^ lipid‐based formulation approach.^318^ On the contrary, the long‐term safety of natural products in reproductive function and EM recurrence profiles should be further elaborated in future studies. Nevertheless, minimal side effects and available as an over‐the‐counter dietary supplement and routine remedies make them preferable to hormonal medicines.

EM is a complex clinical challenge, and recently, more signaling pathways have been identified to contribute to its pathophysiology. EM drugs that target only one receptor have inadequate therapeutic efficiency. However, although multitarget drugs present potent efficacy in suppressing the progression of EM lesions, they also pose a risk of side effects such as binding to undesirable drug targets and bringing off‐target toxicities.^56^ Therefore, designing a drug that targets the appropriate pathways with high selectivity is highly desirable. For this purpose, it is essential to understand the compound‐target pathway‐disease relationships.

## 5 TRADITIONAL CHINESE MEDICINE (TCM)

In the theory of Chinese medicine, EM is defined as a blood stasis syndrome that leads to the formation of endometriotic lesion and other associated symptoms. Stagnation of Qi (energy) is believed to be one of the causes of EM. TCM aims to lessen the chronic pain experienced by women with EM. Therefore, studies on the action mechanism of TCM are focused mainly on the alleviation of inflammation and oxidation. TCM decoctions containing several herbs in different compositions, which are varied according to the condition of the patient, are a combinational approach that can target various pathophysiology. Fang et al.^319^ and Tsai et al.^320^ have identified the decoctions commonly used for treating EM in Taiwan, which included Gui‐Zhi‐Fu‐Ling‐Wan, Dang‐Gui‐Shao‐Yao‐San, Jia‐Wei‐Xiao‐Yao‐San, Shao‐Fu‐Zhu‐Yu‐Tang, and Wen‐Jing‐Tan. The therapeutic efficacy and pathophysiology of TCM in cancer and other diseases have been widely evaluated in vitro and in vivo; however, there are limited studies on the efficacy of TCM for EM.

Most of the herbs exert anti‐inflammatory effects by inhibiting the production of proinflammatory cytokines. Poria has been confirmed to exert antitumor activities against various cancers. It binds to cytokines and effector immune cells to regulate immunity and upregulate apoptosis.^321^ Angelicae Sinensis Radix exerts anti‐inflammatory effects by reducing TNF‐α inflammatory cells.^322^ Ligusticum Rhizoma inhibits inflammation and reduces PGE_2_ production.^323^ Moutan Cortex, Glycyrrhizae Radix, Paeoniae Alba Radix, and Bupleuri Radix suppress proinflammatory cytokines via the NF‐κB signaling pathways.^324^, ^325^, ^326^, ^327^ Paeoniae Alba Radix and Bupleuri Radix also exert such effect via MAPK signaling pathways.

Atractylodis Ovatae Rhizoma exerts antioxidant effect by activating the MAPK cascades and inhibiting the production of radicals by 2,2‐diphenyl‐1‐picrylhydrazyl and catalases, thus inhibiting the activity of free radicals.^328^ Glycyrrhizae Radix and Poria act as radical scavengers against superoxide and hydroxy radicals.^328^, ^329^, ^330^ Ligusticum Rhizoma acts as a reducing agent via the Nrf2 and NF‐κB pathways.^331^

Angelicae Sinensis Radix exerts antiproliferative and proapoptotic effects; it induces mitochondrial‐dependent apoptosis and inhibits the Akt/mTOR pathway.^332^ Atractylodis Ovatae Rhizoma induces apoptosis by upregulating ROS.^333^ Moutan Cortex exerts proapoptotic effects by increasing Bax/Bcl‐2 expression and decreasing MMP via the formation of apoptosome and cytochrome *c*, activation of CASP, and the adenosine monophosphate‐activated protein kinase pathway.^323^ It also induces apoptosis via activation of CASP‐3/‐8.^334^ Paeoniae Alba Radix induces apoptosis via activation of CASP‐3/‐9^293^ and exerts antiproliferative activity via cell cycle arrest and Fas/Fas ligand‐mediated apoptotic pathway.^334^ It also downregulates the antiapoptotic protein Bcl and upregulates the apoptotic proteins Bax and CASP‐3.^335^

TCMs have great potential as multitarget drugs. As TCMs consist of herbal formulas with various combinations of herbs, they have multiple mechanisms of action, which can be beneficial to reduce the concentration of each herb, thus, drug toxicity.^336^ However, the costs and availability vary for different herbs, which limits its acceptability in Western countries at present. Furthermore, there is a lack of clinical management methods to evaluate their clinical effectiveness and standardized regulations of TCM practice.

## 6 CONCLUDING REMARKS AND PERSPECTIVES

This is the first review article combining medicinal research based on EM pathophysiology and the related signaling pathways. Our review revealed the challenges in EM management and the need for various available medical treatment options. Most of the medications prescribed by the FDA to treat EM are hormonal, such as contraceptives, progesterone, and GnRH. However, current hormonal medicines raise a major concern in the case of long‐term treatment. Therefore, new nonhormonal pharmaceuticals with relatively safer and few side effects are urgently needed.

Our aims in this review were to facilitate the research and development of novel treatments for EM based on an understanding of the pathological process. To compare new and old pharmaceuticals, an effective scale to evaluate parameters between different treatments as well as to align outcome measures from preclinical to clinical studies is needed. There is a lack of experimental and clinical evidence to support the effectiveness, pharmacokinetic, and pharmacodynamic profiles of potential drugs in alleviating the pathophysiology of EM, compared with that of drugs already available in the market. Good practices such as the Endometriosis Phenome and Biobanking Harmonization Project, derived by the World Endometriosis Society, can help facilitate a large‐scale collaboration project worldwide.^337^ It is a platform to ensure that the protocol is sufficient and consistent enough to maintain high research quality, datasets are shared to ensure data reproducibility, and results can better support the development of translational medicine. Moreover, multicenter collaboration can increase research visibility and avoid data integrity issues.

The nonhormonal treatments reviewed in this paper were only studied in vitro or in animal models or are still under clinical trials. The drugs mentioned in this review article showed significant efficacy in reducing ectopic endometrium cell viability and endometriotic lesion size; however, severe adverse effects were not elaborated in‐depth. High efficacy and innovative approach do not guarantee final success. Data from legal regulation and patients’ demand for available resources are as important as the pharmacological profile of medicines. In many countries, a new drug must be regulated and approved by the relevant authority before it is launched in the market.^338^ Thus, apart from efficacy and safety data, the medical and financial burden of EM to women have raised the awareness on EM and accelerated the scientific research on this disease, which are key factors considered by R&D investors. To maximize a drug's value and cost‐effectiveness in the market while maintaining its affordability, fulfilling the society's demand, and making scientific advances, modification of lead compound or bioactive compound derived from natural products holds great potential because only the functional groups are modified, whereas the original core structure is conserved.

Considering both the medicinal and commercial perspectives of drug development, there is a huge pressure in the development of a new drug, starting from the synthesis or discovery stage to clinical trial, to proceeding with legal regulations, and to launch in the market. A drug requires 10–17 years of development, with less than 10% success rate to pass clinical trial.^339^

Taking advantage of big data mining, drug repurposing is a strategy to identify new therapeutic use of a drug that is approved or under clinical trial, which comprises 30% of newly FDA‐approved drugs and vaccines.^340^, ^341^ These drugs can bind to the same target owing to the similar pathophysiology of different diseases, or these drugs can have multiple targets and are thus relevant to other diseases.^342^ A repurposed drug offers sufficient preclinical pharmacology profile and safety reports, leading to a greater potential for phase III and IV clinical trials, which can reduce the time of drug development and cost of investment. Nevertheless, some advantages of de novo drug development outweigh the benefits of drug repurposing. A constant influx of chemicals via synthesis or extraction from natural products offers novel medical options for patients. A growing understanding of the pathophysiology of EM favors structure‐based or ligand‐based drug designs, in which by modifying lead compounds based on structure–activity relationships, the efficacy, potency, and selectivity can be compromised. However, a more in‐depth research is needed to study the underlying mechanisms and drug targets to support their potential as new EM treatments.

In conclusion, this review provides an update on the pathophysiology of EM and shows the efficacy of various medicines in treating EM. Increasing attention has been focused on understanding the pathophysiology of EM and the action mechanisms of potential pharmaceuticals; however, many of these are still not completely understood. With this review, we hoped to raise awareness on the missing puzzle pieces and to promote related research that can further advance diagnosis and treatment for better management of EM and improve the quality of women's lives.

## CONFLICT OF INTERESTS

Chi Chiu Wang is an active member of the World Endometriosis Society and an advisor of the Aptorum Group.

## AUTHOR CONTRIBUTIONS

Sze Wan Hung, Ruizhe Zhang, and Chi Chiu Wang participated in research design. Sze Wan Hung participated in data evaluation, extraction and interpretation. Sze Wan Hung, Ruizhe Zhang, and Zhouyurong Tan participated in data validation and in drafting the manuscript. Tao Zhang participated in designing figure 1. Sze Wan Hung, Tao Zhang, Jacqueline Pui Wah Chung and Chi Chiu Wang critically revised the manuscript. All authors approved the final version of the manuscript.
